# Bioinformatics Analysis Reveals E6 and E7 of HPV 16 Regulate Metabolic Reprogramming in Cervical Cancer, Head and Neck Cancer, and Colorectal Cancer through the PHD2-VHL-CUL2-ELOC-HIF-1α Axis

**DOI:** 10.3390/cimb46060370

**Published:** 2024-06-19

**Authors:** Adán Arizmendi-Izazaga, Napoleón Navarro-Tito, Hilda Jiménez-Wences, Adilene Evaristo-Priego, Víctor Daniel Priego-Hernández, Roberto Dircio-Maldonado, Ana Elvira Zacapala-Gómez, Miguel Ángel Mendoza-Catalán, Berenice Illades-Aguiar, Mónica Ascención De Nova Ocampo, Eric Genaro Salmerón-Bárcenas, Marco Antonio Leyva-Vázquez, Julio Ortiz-Ortiz

**Affiliations:** 1 Laboratorio de Biomedicina Molecular, Facultad de Ciencias Químico Biológicas, Universidad Autónoma de Guerrero, Av. Lázaro Cárdenas S/N, Ciudad Universitaria, Colonia La Haciendita, Chilpancingo C.P. 39090, Guerrero, Mexico; adanarizmendi@uagro.mx (A.A.-I.); 09226377@uagro.mx (A.E.-P.); 15168116@uagro.mx (V.D.P.-H.); zak_ana@yahoo.com.mx (A.E.Z.-G.); mamendoza@uagro.mx (M.Á.M.-C.); b.illadesaguiar@gmail.com (B.I.-A.); 2 Laboratorio de Biología Celular del Cáncer, Facultad de Ciencias Químico Biológicas, Universidad Autónoma de Guerrero, Av. Lázaro Cárdenas S/N, Ciudad Universitaria, Colonia La Haciendita, Chilpancingo C.P. 39090, Guerrero, Mexico; nnavarro@uagro.mx; 3 Laboratorio de Investigación en Biomoléculas, Facultad de Ciencias Químico Biológicas, Universidad Autónoma de Guerrero, Av. Lázaro Cárdenas S/N, Ciudad Universitaria, Colonia La Haciendita, Chilpancingo C.P. 39090, Guerrero, Mexico; wences2009@hotmail.com; 4 Laboratorio de Investigación Clínica, Facultad de Ciencias Químico Biológicas, Universidad Autónoma de Guerrero, Av. Lázaro Cárdenas S/N, Ciudad Universitaria, Colonia La Haciendita, Chilpancingo C.P. 39090, Guerrero, Mexico; rdircio@uagro.mx; 5 Escuela Nacional de Medicina y Homeopatía, Programa Institucional de Biomedicina Molecular, Instituto Politécnico Nacional, Guillermo Massieu Helguera No. 239 Col. Fracc. La Escalera-Ticomán, Ciudad de Mexico C.P. 07320, Mexico; mdenova@ipn.mx; 6 Departamento de Biomedicina Molecular, Centro de Investigación y de Estudios Avanzados del Instituto Politécnico Nacional, Ciudad de México C.P. 07360, Mexico; eric.salmeron@cinvestav.mx

**Keywords:** HPV 16, E6, E7, HIF-1α, metabolic reprogramming

## Abstract

Human papillomavirus 16 (HPV 16) infection is associated with several types of cancer, such as head and neck, cervical, anal, and penile cancer. Its oncogenic potential is due to the ability of the E6 and E7 oncoproteins to promote alterations associated with cell transformation. HPV 16 E6 and E7 oncoproteins increase metabolic reprogramming, one of the hallmarks of cancer, by increasing the stability of hypoxia-induced factor 1 α (HIF-1α) and consequently increasing the expression levels of their target genes. In this report, by bioinformatic analysis, we show the possible effect of HPV 16 oncoproteins E6 and E7 on metabolic reprogramming in cancer through the E6-E7-PHD2-VHL-CUL2-ELOC-HIF-1α axis. We proposed that E6 and E7 interact with VHL, CUL2, and ELOC in forming the E3 ubiquitin ligase complex that ubiquitinates HIF-1α for degradation via the proteasome. Based on the information found in the databases, it is proposed that E6 interacts with VHL by blocking its interaction with HIF-1α. On the other hand, E7 interacts with CUL2 and ELOC, preventing their binding to VHL and RBX1, respectively. Consequently, HIF-1α is stabilized and binds with HIF-1β to form the active HIF1 complex that binds to hypoxia response elements (HREs), allowing the expression of genes related to energy metabolism. In addition, we suggest an effect of E6 and E7 at the level of PHD2, VHL, CUL2, and ELOC gene expression. Here, we propose some miRNAs targeting PHD2, VHL, CUL2, and ELOC mRNAs. The effect of E6 and E7 may be the non-hydroxylation and non-ubiquitination of HIF-1α, which may regulate metabolic processes involved in metabolic reprogramming in cancer upon stabilization, non-degradation, and translocation to the nucleus.

## 1. Introduction

High-risk HPVs (HPV-HR) has been associated with approximately 5% of all human cancers worldwide [1], and it is associated with HPV 16 [2], being found in 61% of cervical cancer, 95% of oropharyngeal cancer, 75% of anal cancer, 40–80% of colorectal cancer, and 60% of penile cancer cases [3,4,5,6]. The oncogenic potential of HPV 16 is due to the ability of E6 and E7 oncoproteins to promote alterations associated with cell transformation [7]. E6 and E7 of HPV 16 have been observed to affect metabolic reprogramming by favoring the expression of some transporters and enzymes of glycolysis, such as GLUT1, HKII, PFK, ENOA, PKM2, and LDHA [8]. In contrast, oxidative phosphorylation (OXPHOS) is affected by HPV 16 by modulating mitochondrial structure, release, and increase in reactive oxygen species (ROS); inactivation of mitochondrial complex III, ATP synthase, and decreasing levels of glutathione (GSH); and superoxide dismutase 1 and 2 (SOD1 and SOD2) [8].

Metabolic reprogramming is now considered a hallmark of cancer [9], necessary for growth, cell division, migration, and metastasis [10,11]. In cancer, cells produce energy mainly through aerobic glycolysis rather than oxidative phosphorylation (OXPHOS), a process known as the “Warburg effect” [12]. This process favors obtaining energy from adenosine 5′-triphosphate (ATP) necessary in cancer progression [13].

It has been reported that O_2_ concentration is reduced in many human cancers compared to normal tissue [14]. In cancer, decreased O_2_ pressure (pO_2_) is associated with an increased risk of tumor invasion and metastasis [15]. Low O_2_ concentration favors the activation of HIF-1, a critical transcription factor composed of alpha and beta subunits (HIF-1α and HIF-1β) [16]. Both subunits form the active HIF-1 dimer that binds at specific sites called hypoxia response elements (HRE) in DNA to transcribe its target genes [17], primarily genes related to glucose metabolism and pH regulation [18] such as transferrin (*TF*); glucose transporter 1 (*GLUT1*); hexokinase 2 (*HK2*); phosphofructokinase (*PFK*); phosphoglycerate kinase (*PGK*); enolase 1, 2, and 3 (*ENO1, 2, 3*); aldolase C (*ALDO-C*); pyruvate kinase M2 (*PKM2*); pyruvate dehydrogenase kinase 1 (*PDK1*); lactate dehydrogenase A *(LDH-A*); carbonic anhydrase IX (*CAIX*); 6-phosphofructo-2-kinase/fructose-2,6-bisphosphatase 4 (*PFKFB4*); monocarboxylate transporter 4 (*MCT 4*); and sodium–hydrogen exchanger *(NHE*) [18]. Under normal O_2_ conditions, the HIF-1α subunit is continuously expressed. However, it is hydroxylated at proline residues 402 and 564 (P402 and P564) and asparagine 803 (N803) by dioxygenases, prolyl-4-hydroxylases (PHD), and asparaginyl hydroxylase or HIF-1α inhibitory factor (FIH-1), respectively [19]. When HIF-1α is hydroxilated, is recognized by the elongation and ubiquitination complex VCB-CR constituted by von Hippel–Lindau (VHL), Elongin C (ELOC), Elongin B (ELOB), Cullin 2 (CUL2), ring box protein 1 (RBX1), E2 ubiquitin-conjugating enzyme (E2), and spermidine/spermine protein N (1)-acetyltransferase 2 (SSAT2) to be subsequently degraded by the 26S proteasome [18,19]. Elongin B and Elongin C participate as adaptor molecules that bind the substrate recognition subunit of the VCB-CR complex (pVHL, which binds substrates through its β domain) to heterodimers of CUL2, and RBX1 is stabilized by associating with Elongins B and C and, in turn, Elongins B and C are stabilized by their interactions with each other and with pVHL. The formation of this elongation–ubiquitination complex (VCB-CR) is stabilized for subsequent degradation of HIF-1α. Under hypoxia conditions, PHD activity is impaired, and the formation of the complex (VCB-CR) is prevented, recognition of HIF-1α is impaired, HIF-1α is stabilized, and a hyperglycolytic phenotype is generated in cancer cells [20]. High-risk HPV infection has been reported to lead to aberrant expression of miRNAs [21,22]. Some of these miRNAs negatively regulate multiple metabolic genes, involved in the tricarboxylic acid (TCA) cycle, aerobic glycolysis, de novo synthesis of fatty acids, and altered autophagy, which allow tumor cells to survive in adverse conditions such as the absence of nutrients and oxygen. In addition, miRNAs may target signaling molecules such as HIF-1 that facilitate metabolic adaptation in tumor cells [23]. Interestingly, some miRNAs have been reported to target *HIF1A*, *VHL*, *CUL*, *RBX1*, and *ELOC* [24,25,26].

It has been reported that E6 plays a crucial role in regulating the Warburg effect by blocking the degradation of HIF-1α and, consequently, its stability and nuclear localization [27]. In contrast, E7 increases the stability and expression of HIF-1α under hypoxia conditions [28,29] by inhibiting the expression of the hepatic kinase B1 (LKB1) tumor suppressor responsible for repressing HIF-1α expression [30] and by inhibiting Ras-related associated with diabetes (RRAD), which increases the levels of the p65 subunit of NF-kB, the transcription factor that promotes HIF-1α expression [31]. Here, we found that HPV oncoproteins E6 and E7 affect metabolic reprogramming through the action of miRNAs by inhibiting the expression of VCB-CR complex proteins and consequently favoring the stability and activity of HIF-1α. We also validate the information using different bioinformatics databases and propose the possible mechanism through which E6 and E7 could participate in the stability and translocation of HIF-1α to the nucleus and the formation of the active HIF-1 complex. Furthermore, we propose the implications of active HIF-1 formation in metabolic reprogramming.

## 2. Materials and Methods

### 2.1. Analysis of HIF1A, VHL, and PHD2 (EGLN1) Expression in Cervical Cancer Patient Samples Using TCGA, GTEx, GEO, and HPA Databases

HIF-1A expression was analyzed in normal tissue samples, cervical cancer (CC), head and neck squamous cell carcinoma (HNSC), and colorectal cancer (CRC), as well as colon adenocarcinoma (COAD) and rectal adenocarcinoma (READ), using The Cancer Genome Atlas (TCGA) and The Genotype-Tissue Expression (GTEx) datasets, which use the Gene Expression Profiling Interactive Analysis (GEPIA) database. GEPIA is a newly developed web server for analyzing RNA sequencing expression data from RNA-Seq datasets of 9736 tumors and 8587 normal samples from the TCGA and GTEx projects [32]. The GSE67522 (Illumina HumanHT-12 V4.0 expression beadchip) [33] and GSE9750 ([HG-U133A] Affymetrix Human Genome U133A Array) [34] from the Gene Expression Omnibus (GEO) database [35,36] were used to determine the expression of *HIF1A*, *VHL*, and *PHD2* (*EGLN1*) in samples from HPV-positive and -negative CC patients and non-malignant samples. Samples were from CC patients with HPV genotypes 16, 18, and 45; other genotypes; and normal cervical samples. To determine the expression of *HIF1A* by HPV 16 E6 effect, the GSE73761 dataset ([HTA-2_0] Affymetrix Human Transcriptome Array 2.0 [transcript (gene) version]) [37] from GEO was used, which includes transfection of the HPV 16 E6 gene (E-prototype) and HPV 16 E6 variants AA-a, AA-c, E-A176/G350, E-C188/G350, and E-G350 in C33-A cells (HPV-negative cervical cancer cells). Dataset GSE67522 consists of 31 cervical cancer samples, 11 HPV-negative and 20 HPV-positive samples, and 11 non-malignant samples. The GSE9750 dataset consists of 21 normal cervical samples, 19 HPV 16-positive CC samples, 3 HPV 18-positive samples, 3 HPV 45-positive samples, 6 samples with other HPV types, and 2 HPV-negative CC samples. The GSE73761 dataset is composed of 2 samples from each group, C-33A cells transfected with the empty vector and transfected with the E6 variants of HPV 16, E-protype, AA-a, AA-c, E-A176/G350, E-C188/G350, and E-G350. 

HIF-1α protein levels were analyzed by immunohistochemistry assays in CC, HNSC, CRC, and normal tissue samples from the cervix, head and neck, colon, and rectum, which were obtained from the Human Protein Atlas (HPA) database. The HPA is a repository that aims to map all human proteins in cells, tissues, and organs through an integration of various omics technologies, including antibody-based imaging, mass-spectrometry-based proteomics, and transcriptomics of cancer patients for nearly 8000 cancer patients representing 17 major cancer types [38]. Representative images of cancer and normal tissues were randomly selected.

### 2.2. Survival Analysis

Overall survival (OS) analyzes were performed from the TCGA dataset using the Kaplan–Meier Plotter database (https://kmplot.com/analysis/ accessed on 28 August 2023). The Kaplan–Meier Plotter is an online survival analysis tool, which performs all real-time calculations of more than 35,000 samples from 21 tumor types. Gene expression as well as overall and relapse-free survival information was downloaded from the GEO, EGA, and TCGA databases [39]. Survival curves were estimated using the Kaplan–Meier estimator. The survival curves were compared with the log-rank test, the means were calculated for all the databases, the FC values were considered below 1, and all the subtypes were included in the analysis and the cellular content. Data were analyzed for CC, HNSC, and rectal cancer (READ) using the pan-cancer expression option. A total of 304 patients from the database repository were analyzed, corresponding to patients diagnosed with CC, 500 with HNSC and 165 patients with READ.

### 2.3. Interactome Analysis of E6, E7, and HIF-1α

The interaction analysis between E6, E7, and HIF-1α was performed on data from the BioGRID 4.4 repository (https://thebiogrid.org/ accessed on 28 August 2023). BioGRID contains 1,740,000 genetic and protein interactions from model organisms and humans, culled from high-throughput datasets and individually focused studies derived from over 70,000 publications in the primary literature [40,41]. We used access links for E6 (https://thebiogrid.org/4263557 accessed on 28 August 2023) and E7 (https://thebiogrid.org/4263558 accessed on 28 August 2023) of HPV 16 and HIF-1α (https://thebiogrid.org/109338 accessed on 28 August 2023). The interactome was generated in Cytoscape 3.8.2 software.

### 2.4. Interaction Sequence Analysis

Interaction sequence analysis was performed using the IntAct EMBL-EBI (https://www.ebi.ac.uk/intact/ accessed on 22 September 2023). IntAct provides a free and open source database system and analysis tools for molecular interaction data, stores 134,556 and 808,073 interaction from 23,366 publications [41,42,43] and InterPro EMBL-EBI (https://www.ebi.ac.uk/interpro/ accessed on 26 September 2023). InterPro is a resource that provides functional analysis of protein sequences by classifying them into families and predicting the presence of important domains and sites, it integrates information from 13 databases: CATH, CDD, HAMAP, MobiDB Lite, Panther, Pfam, PIRSF, PRINTS, Prosite, SFLD, SMART, SUPERFAMILY and NCBIfam [44,45].

### 2.5. PDZ Domain Search

PDZ domain search was performed in the PDZscape database (http://www.actrec.gov.in:8080/pdzscape/ accessed on 5 October 2023). PDZscape encompasses the complete information available on 58,648 PDZ-containing proteins with their known and putative binding partners in one platform, with a unique integration of prominent databases, including NCBI, UniProtKB, Swiss-Prot, Pubmed, PDB, STRING, IntAct, KEGG, Pfam, and the Protein Mutant Database, providing detailed information on the PDZ interactome in addition to the custom BLAST option [46]. 

### 2.6. Search for miRNAs

The search for miRNAs was performed using the TargetScan (http://www.targetscan.org/vert_72/ accessed on 8 November 2023) [47], miRWalk (http://mirwalk.umm.uni-heidelberg.de/ on 8 November 2023) [35], and miRDB (http://mirdb.org/ on 10 Novembre 2023) [48]. We used VENNY 2.0 (https://bioinfogp.cnb.csic.es/tools/venny/ on 16 November 2023) to obtain the common miRNAs in the three databases and possible candidates.

### 2.7. miRNA Expression Analysis

The miRNA gene expression data were obtained from the GSE158659 dataset [49] from the GEO database and were used to validate the expression of miRNAs in HPV-positive and HPV-negative cell lines. The GSE158659 dataset consists of 14 cancer cell lines, 3 HPV 16-positive head and neck squamous cell carcinoma (HNSCC) cell lines (SCC-154, SCC-090, SCC-047), 4 HPV-negative HNSCC cell lines (UPCI-017, UPCI-068, SCC-4, SCC-1), 2 HPV-negative cervical squamous cell carcinoma (CSCC) cell lines (HT-3, C-33A), 2 HPV 16-positive CSCC cell lines (SiHa, Ca Ski), and 3 HPV 18-positive CSCC lines (C-4 I, HeLa, SW756). Expression of miRNAs was performed by sequencing on the Illumina HiSeq 3000 platform (Homo sapiens). 

### 2.8. Function Analysis 

Function analysis was performed from the list of proteins that have physical, protein/gene, and chemical interaction with HIF-1α, E6, and E7 of HPV 16 (https://thebiogrid.org/109338 on 5 December 2023) in the GENEONTOLOGY (http://geneontology.org/ on 5 December 2023). Gene Ontology provides a computational representation of the current scientific knowledge about the functions of genes (or, more properly, the proteins and non-coding RNA molecules produced by genes) of many different organisms, from humans to bacteria [50]. PANTHER (http://pantherdb.org/ on 5 December 2023), is a comprehensive, annotated “library” of phylogenetic trees of gene families, providing a stable substrate for annotations of protein properties as subfamily and function, enriched with experimental validation [51] GOTERMFINDER (https://go.princeton.edu/cgi-bin/GOTermFinder on 5 December 2023) comprises a set of object-oriented Perl modules for accessing Gene Ontology (GO) information and evaluating and visualizing the collective annotation of a gene list in GO terms. It can be used to draw conclusions from microarrays and other biological data [52]. The Metascape (https://metascape.org/gp/index.html#/main/step1 on 14 December 2023) database is a web portal designed to provide a comprehensive gene list annotation and analysis resource for experimental biologists, combining functional enrichment, interactome analysis, gene annotation, and membership search to leverage over 40 independent knowledge bases within an integrated portal [53]. The processes and functions were integrated into the metabolic pathway network generated in esyN (https://esyn.rosalind.kcl.ac.uk/ on 14 December 2023).

### 2.9. Statistical Data

Protein interaction analyses are shown to have experimental validation. PDZ domain data were accepted from the score, percent positivity, and percent identity. The PDZ domain of VHL shares a score of 37.7 bits (86), 50% positivity, and 35% identity compared to that of the PDZscape database. The reliability parameters were <=0.001 for GOTERMFINDER and <0.001 for GO and PANTHER. For Metascape, the reliability values were obtained by −Log(p). Candidate miRNAs were obtained from the parameters: Position in the UTR, seed match (8 mer), and Context score percentile 62–99. HIF-1A gene expression was log2 transformed (TPM + 1), differences were calculated using a one-way ANOVA test, and a *p*-value of <0.05 was considered statistically significant. Statistical differences of VHL, PHD2 (EGLN1), CUL2, ELOC, and miRNA expression were calculated using one-way ANOVA and Student’s *t*-test using GEPIA and GEO databases, respectively. A value of * *p* < 0.05 was considered significant.

## 3. Results

### 3.1. HIF1-α Expression in Cancer

**Figure 1:**
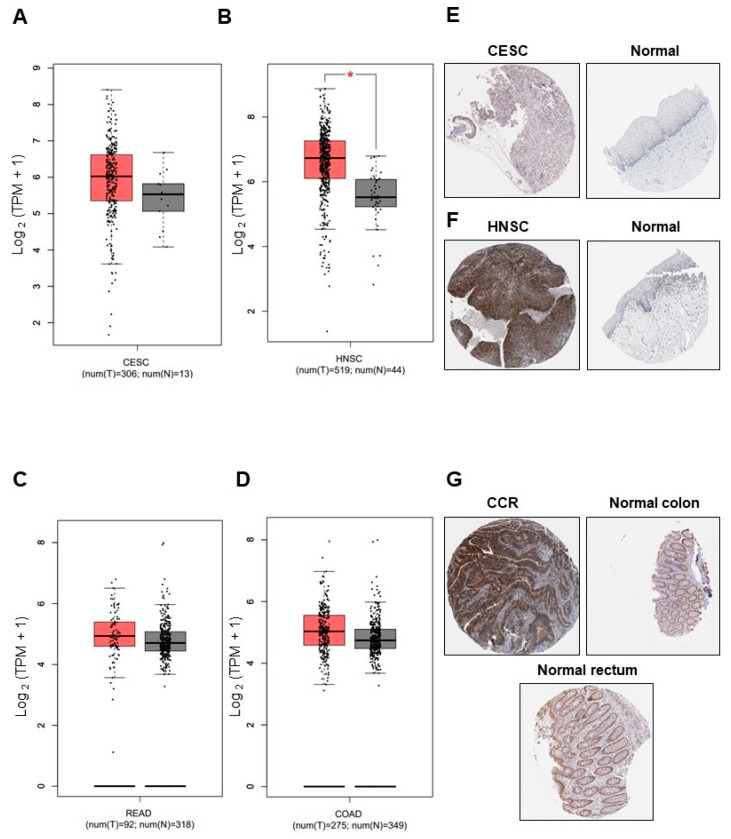
HIF1-α expression was increased in CC, HNSC, and CRC cancer. *HIF1A* messenger expression in CC (**A**), HNSC (**B**), CRC (**C**,**D**), and TCGA normal tissue samples. One-way ANOVA, * *p* < 0.05. HIF-1α protein expression level in CC (**E**), HNSC (**F**), CRC (**G**), and normal tissue from the THPA database. A representative image is presented in each case.

To determine the analysis of *HIF1A* messenger expression in TCGA and GTEx using the GEPIA database showed an increase in CC, HNSC, and CRC (COAD and READ) compared to normal tissue samples. *HIF1A* increases its expression in CC, HNSC, COAD, and READ, although the increase was only significant in HNSC (Figure 1A–D). Protein level analysis of HIF1-α was performed in the HPA database. According to the *HIF1A* messenger expression data obtained in GEPIA, high levels of HIF-1α protein were found in CC, HNSC, COAD, and READ compared with normal tissue (Figure 1E–G). We did not find HIF1-α expression levels in penile cancer.

**Figure 2:**
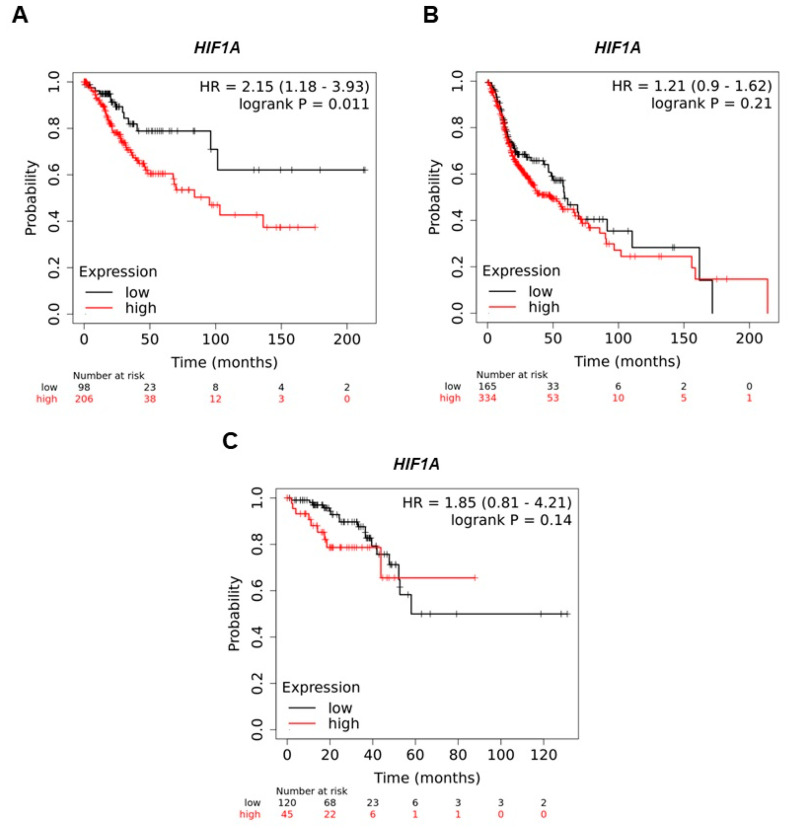
Analysis of overall survival (OS) by *HIF1A* expression in patients with CC, HNSC, and CRC cancer. The data show the probability of survival of patients who express high *HIF1A* expression in patients with CC (**A**), HNSC (**B**), and rectal (**C**) cancer, for 200 and 120 months, during which time, the levels of *HIF1A* were studied. The red lines show high levels, and the gray color shows low levels of gene expression. The numbers below the graphs indicate the number of patients during the initial expression analysis—50, 100, 100, 150, and 200 or 20, 40, 60, 80, 100, and 120 months. *p* < 0.05 was considered statistically significant.

To determine if high *HIF1A* expression is involved in the survival of CC, HNSC, and CRC cancer patients, OS analyzes were performed using the Kaplan–Meier Plotter database, accessed 28 August 2023. High *HIF1A* expression is associated with shorter OS in patients diagnosed with CC, HNSC, and rectal cancer (Figure 2A–C). However, the data were only significant in CC. It was observed that there were 2.15, 1.21, and 1.85 greater risks of dying in patients diagnosed with CC (A), HNSC (B), and rectal cancer (C), when they express high levels of *HIF1A*, respectively, compared to patients with low levels of *HIF1A* expression. For the OS analysis, we did not find the expression levels for penile cancer and colon cancer.

### 3.2. HIF-1α Expression in HPV-Positive and HPV-Negative Cervical Cancer Samples

**Figure 3:**
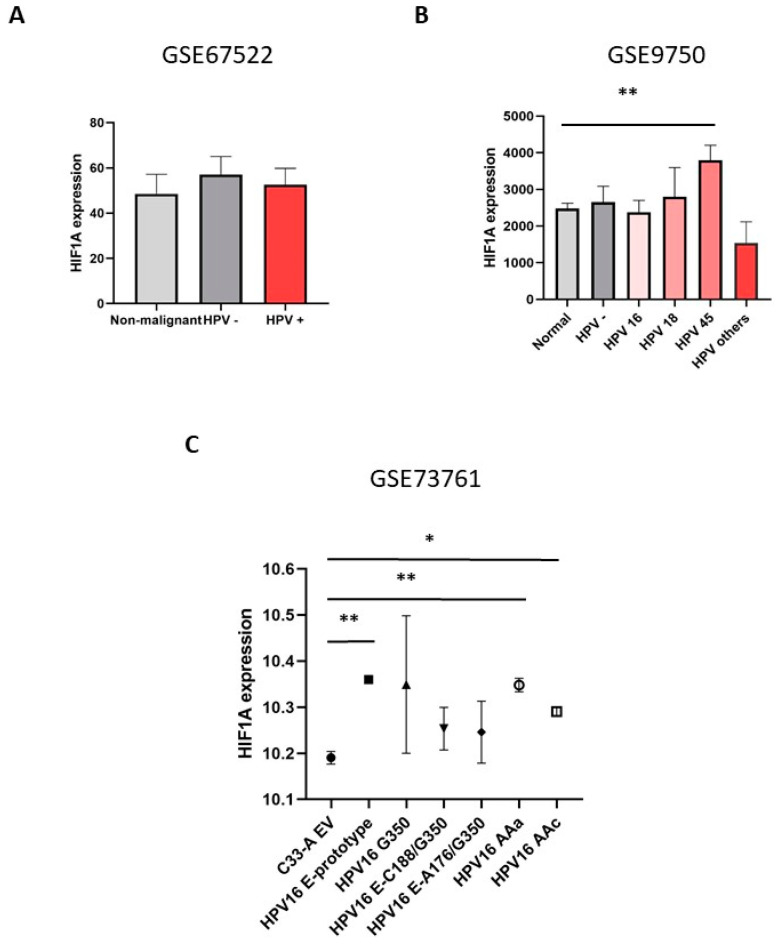
*HIF1A* expression in HPV-positive and HPV-negative CC samples. (**A**) *HIF1A* expression from dataset GSE67522, including non-malignant patient samples as well as HPV-negative (HPV−) and HPV-positive (HPV+) CC samples. (**B**) *HIF1A* expression using the GSE9750 dataset, including normal samples as well as CC samples positive for HPV genotypes 16, 18, and 45, and other genotypes. (**C**) *HIF1A* expression from the GSE73761 dataset, cervical cancer cell samples from the C-33A cervical cancer cell line (HPV-negative tumor cells), E6-transfected C-33A cells, and E6 HPV 16 variants (G350, E-C188/G350, E-A176/G350, AAa, and AAc) were included (The symbols represent the different variants of HPV 16). Fold changes of *HIF1A* were analyzed in GraphPad Prism 8 software. * *p* < 0.05, ** *p* < 0.01.

Cervical cancer is a unique tumor model to understand the biological role of E6 and E7 viral oncoproteins in carcinogenesis [22]. To determine whether HIF1A expression in CC might be downregulated in the presence of HPV, expression analysis was performed from the GSE67522, GSE9750, and GSE73761 dataset. The samples of CC patients with and without HPV; patients with the high-risk HPV genotypes HPV 16, 18, 45, and other HPV genotypes; and from C-33A tumor cells (HPV-negative tumor cells) transfected with E6 of HPV 16 and its variants were analyzed. *HIF1A* expression levels showed no statistically significant differences in samples from HPV-positive and HPV-negative patients compared with normal cervical tissue (Figure 3A). Interestingly, *HIF1A* expression was found to be higher and statistically significant in patients with HPV genotype 45 compared to patients with genotypes 16 and 18 (Figure 3B). However, *HIF1A* levels did not increase in the presence of HPV 16. Interestingly, overexpression of HPV 16 E6 (E-prototype) and HPV 16 E6 variants (AAa and AAc) were found to significantly increase *HIF1A* expression in CC C-33A cells (Figure 3C).

Moreover, increased expression of *HIF1A* was found to be related to the presence of HR-HPV and to the overexpression of E6 of HPV 16.

### 3.3. Analysis of the E6, E7, and HIF-1α Interactome and Target Functions of HIF-1α Targets

To determine the possible effect of E6 and E7 oncoproteins on HIF1-α overexpression in CC, HNSC, and CRC where HPV 16 is a preponderant risk factor for its development, an interactome analysis was performed in the Bio GRID 4.4 database between the E6-E7-HIF1-α axis by integrating the interactomes in Cytoscape 3.8.2 to search for common nodes between in E6, E7, and HIF1-α.

**Figure 4:**
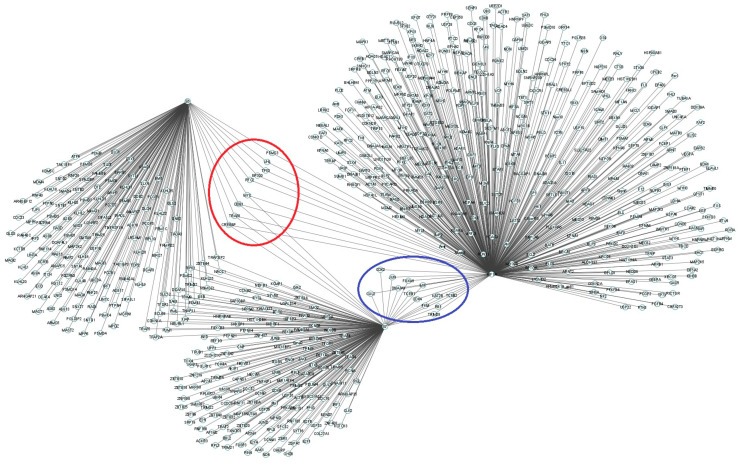
Interactome of the E6-E7-HIF-1α axis. Proteins that interact with E6, E7, and HIF-1α are shown. The proteins that showed interaction with E7-HIF-1α are shown in the blue circle. In the red circle, the proteins that had interaction with E6-HIF-1α are shown. Proteins that had interaction with E6-E7-HIF-1α can also be seen. The interactome was generated in Cytoscape 3.8.2 software.

E6 and HIF1-α were found to have interaction in common with 26S proteasome regulatory subunit 6A (PSMC3), Replication factor C subunit 2 (RFC2), Histone acetyltransferase p300 (EP300), Cellular tumor antigen p53 (TP53), and von Hippel–Lindau tumor suppressor (VHL) (Figure 4, red circle). In contrast, E7 and HIF-1α have interaction with histone-lysine N-methyltransferase SETD1B-like protein (TRM28), Retinoblastoma-associated protein (RB1), Pyruvate Kinase M1/2 (PKM1/2), Lysine Acetyltransferase 2B (KAT2B), Cyclin-dependent kinase 4 (CDK4), Macrophage migration inhibitory factor (MIF), Forkhead box protein M1 (FOXM1), DnaJ homolog subfamily A member 3, mitochondrial (DNAJA3), JUN, Cyclin-dependent kinase 2 (CDK2), Elongin C (TCEB1), Elongin B (TCEB2), and Cullin 2 (CUL2) (Figure 4, blue circle). Moreover, together E6, E7, and HIF-1α showed interaction with CREEBBP, TRAF6, DDB1, and MYC (Figure 4).

To determine the functions of the molecular targets of HIF-1α, a search for the IDs of the 1218 targets mentioned above was performed in the UniProt database. The functions are presented in Table S1. Genes encoding proteins that show interaction with the E6-E7-HIF-1α axis related to HIF-1α stability were found. Based on the functions of the proteins encoded by these genes, it was observed that the ubiquitin ligase VHL has interaction with E6 and HIF-1α and that members of the E3 ubiquitin ligase complex Elongin C, Elongin B, and Cullin 2 (CUL2) have interaction with E7 and HIF-1α.

### 3.4. Interaction Sequence Analysis

**Figure 5:**
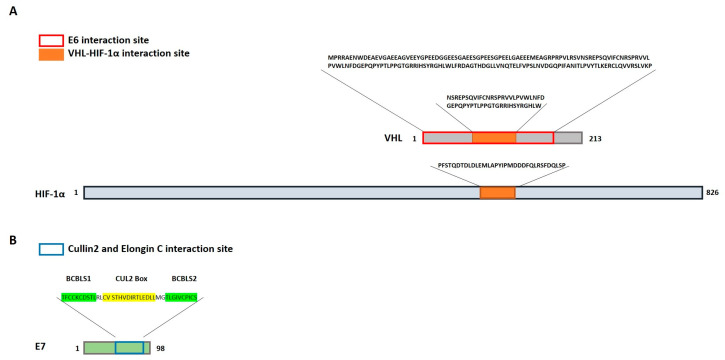
Sequence analysis on the interaction of E6-E7-HIF-1α. (**A**) E6 bound with VHL in the region interacting with HIF-1α. The red box represents the interaction sequence of E6 with VHL. The orange boxes represent the interaction sequences between VHL and HIF-1α. (**B**) CUL2 and ELOC interacted with E7 through their carboxy-terminal domain. The blue box represents the interaction region of CUL2 and ELOC. Letters marked in green represent the interaction sequence of ELOC, and letters in yellow represent the interaction sequence of CUL2 with E7.

In the interaction sequence analysis using the IntAct EMBL-EBI and InterPro EMBL-EBI databases, it was observed that E6 binds to VHL in the region comprising the amino acid residues interacting with HIF-1α (Figure 5A) [54]. On the other hand, E7 binds to CUL2 and ELOC through the C3 domain located in the carboxy-terminal region (Figure 5B) [55,56]. ELOC binds to E7 through BCBLS boxes 1 and 2 (Figure 5B, sequence in green color), and CUL2 binds through the CUL2 box (Figure 5B, sequence in yellow color) [56]. Interestingly, no interaction of E6 and E7 with HIF-1α was observed in the databases used, although reports indicate an interaction [57].

### 3.5. PDZ Domain Analysis

**Figure 6:**
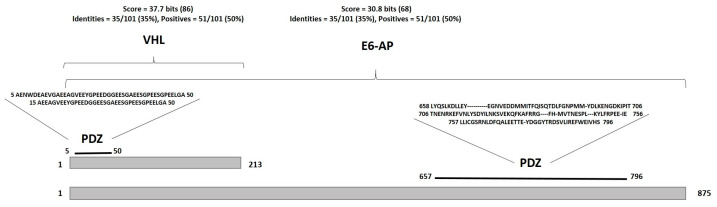
E6 of HPV 16 was able to interact with VHL through a PDZ domain. E6 was able to bind to the sequence located from amino acids 5 to 50 of VHL. The computational parameters are depicted at the top of VHL. The predicted PDZ domain in VHL had a score of 37.7, an identity of 35%, and a positivity of 50% compared to the consensus PDZ of the program. The PDZ of the E6AP protein was located between amino acid positions 657 to 796; the computational parameters are shown at the top of the protein name. The PDZ of E6AP showed a score of 30.8, 35% identity, and 50% positivity. Proteins are shown in gray boxes, and the numbers represent protein size and domain position. PDZ above the line represents the domain position and, above it, the amino acid sequence.

The E6 oncoprotein of high-risk HPVs has the PDZ-binding motif (PBM), a specific sequence located at the carboxy-terminal end that interacts with the PDZ domain of its target proteins [58]. Based on the above, an analysis was performed in the PDZscape database to identify whether VHL and HIF-1α contain a sequence in their structure that could function as a PDZ domain. The data show that VHL has a PDZ domain that, compared with the consensus PDZ in the database, shares a score of 37.7 bits (86), a positivity of 50%, and an identity of 35%; however, in HIF-1α, no PDZ domain was found (Figure 6). Comparing the PDZ domain found in VHL with the PDZ domain of the ubiquitin ligase E6AP, we discovered that the PDZ score of VHL was higher than that of PDZ of E6AP. On the other hand, the identity and positivity were identical in the PDZ of both proteins (Figure 6). These data suggest that E6 could bind to VHL through interaction with the predicted PDZ domain in VHL located between amino acids 5 to 50, located in the binding region of VHL with HIF-1α (amino acids 1 to 172, Figure 5A).

### 3.6. Search for miRNAs That Regulate the Expression of the PHD2 (EGLN1), VHL, CUL2, and ELOC Axis

**Figure 7:**
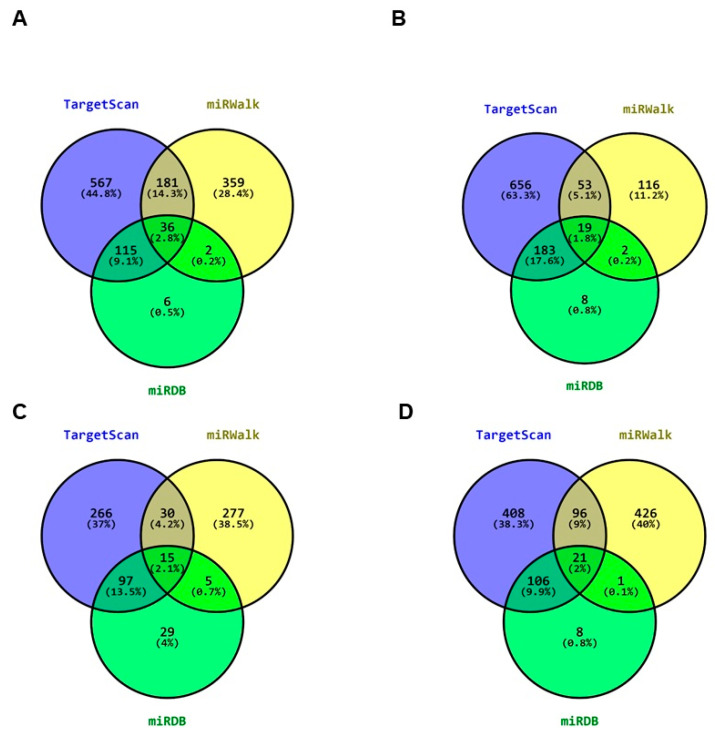
VENN diagram of miRNAs and common miRNAs regulating VHL, PHD2, CUL2, and ELOC mRNA. (**A**) Analysis for VHL, (**B**) analysis for PHD2, (**C**) analysis for CUL2, and (**D**) analysis for ELOC. In the blue circle, the number of miRNAs in the TargetScan database is represented (899 for VHL, 911 for PHD2, 410 for CUL2, and 631 for ELOC). The yellow circle represents the miRNAs in the miRWalk database (578 for VHL, 190 for PHD2, 327 for CUL2, and 544 for ELOC). In the green circle, the number of miRNAs in the miRDB database is represented (159 for VHL, 212 for PHD2, 146 for CUL2, and 136 for ELOC). In common, 36 miRNAs bind to VHL mRNA, 19 to PHD2 mRNA, 15 to CUL2 mRNA, and 21 to ELOC mRNA. The percentage is shown in each of the data obtained.

One mechanism of regulation of gene expression in cancer is known to be generated through miRNAs. The miRNAs are small non-coding RNA molecules of 21 to 25 nucleotides (nt) in length, which specifically bind to target sites in the 3′ untranslated region (UTR) of mRNAs, resulting of mRNA degradation, leading to its non-translation into a protein [59]. The E6 and E7 oncoproteins of HPV 16 lead to aberrant miRNA expression; a large number of these miRNA-containing genes are downstream mRNA targets and are crucial in normal cell maintenance [22,60]. To determine whether E6 and E7 of HPV 16 could alter the VHL-HIF-1α axis at the miRNAs, a search for potential miRNAs targeting *PHD2*, *VHL*, *CUL2*, and *ELOC* mRNA was performed. TargetScan, miRWalk, and miRDB databases were used. Using VENNY 2.0, 36 miRNAs were found in the three databases targeting *VHL* mRNA (Figure 7A), 19 for *PHD2* mRNA (Figure 7B), 15 for *CUL2* mRNA (Figure 7C), and 21 for *ELOC* mRNA (Figure 7D). Of the miRNAs shared by the three databases for each *PHD2*, *VHL*, *CUL2*, and *ELOC* transcript, eight miRNAs were obtained for *VHL*, five for *PHD2*, five for *CUL2*, and seven for *ELOC*, considering the highest confidence parameters in TargetScan (Table 1). Of the miRNAs that regulate VHL mRNA, hsa-miR-155-3p and hsa-miR-143-3p have experimental reports demonstrating that E6 and E7 increase their expression. Likewise, experimental reports indicate that HPV alone can increase the expression of hsa-miR-508-3p [61,62,63].

Experimental reports demonstrated the regulation of *PHD2* mRNA by increasing hsa-miR-182-5p in the presence of E7 [64,65]. On the other hand, for the miRNAs that regulate *CUL2*, experimental data were found demonstrating that the expression of hsa-miR-6129 is associated with the presence of HPV 16 and that the seed region of hsa-miR-297 is found in the HPV 16 genome [66,67]. No experimental basis was found for the miRNAs possibly regulating *ELOC* (Table 1). To validate whether the expression of miRNAs obtained from the bioinformatics analysis increase with the presence of HPV, we analyzed the expression of common miRNAs in the three databases for each transcript *PHD2*, *VHL*, *CUL2*, and *ELOC* with the highest confidence parameters (Table 1) from the GSE158659 dataset of HPV-positive (SCC-154, SCC-090, SCC-047, SiHa, Ca Ski, C-4 I, HeLa, SW756) and HPV-negative (UPCI-017, UPCI-068, SCC-4, SCC-1, HT-3, C-33A) CSCC and HNSCC cell lines.

**Figure 8:**
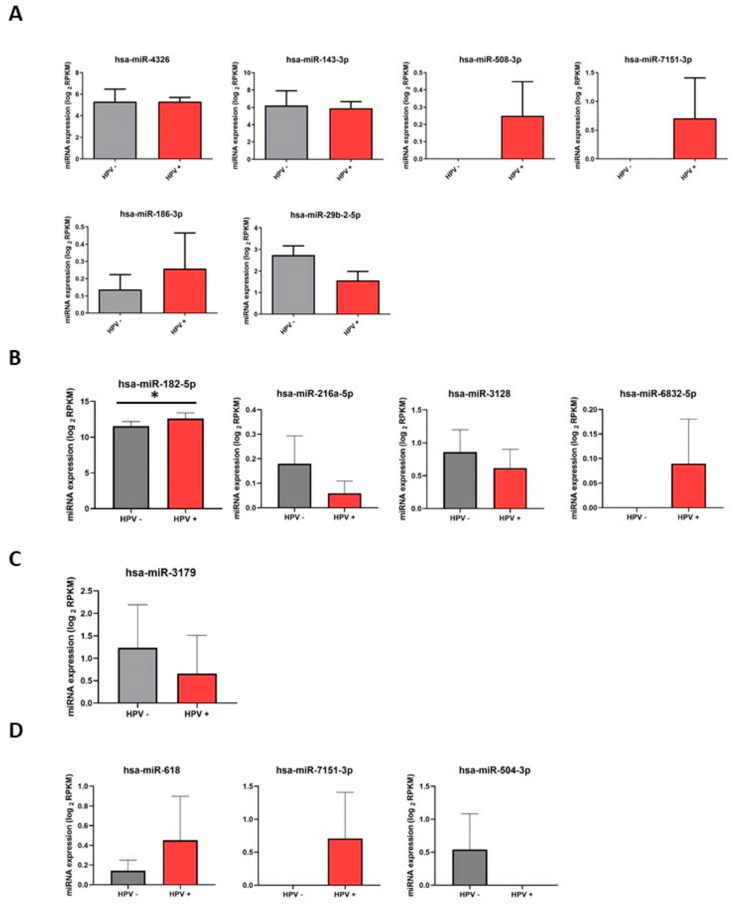
Expression of miRNAs involved in VCB-CR, *VHL*, *PHD2*, *CUL2*, and *ELOC* axis expression in HPV-positive and HPV-negative CSCC and HNSCC cell lines. (**A**) Expression of miRNAs targeting the *VHL* transcript, (**B**) *PHD2*, (**C**) *CUL2*, and (**D**) *ELOC*. Expression of miRNAs was obtained from the GSE158659 dataset. The expression of miRNAs is represented in Log2 RPKM (reads per kilobase million). Expression of miRNAs was analyzed in GraphPad Prism 8 software. Statistical significance: * *p* < 0.05.

We observed high levels of expression of hsa-miR-508-3p, hsa-miR-7151-3p, and hsa-miR-186-3p miRNAs targeting the *VHL* transcript in HPV-positive cells (Figure 8A) compared with HPV-negative cells. Notably, for the miRNAs targeting the *PHD2* (*EGLN1*) transcript hsa-miR-6832-5p and hsa-miR-182-5p, expression levels were high in HPV-positive cells compared to HPV-negative cells, and expression levels were statistically significant for hsa-miR-182-5p (Figure 8B). We also observed high levels of expression of hsa-miR-508-3p, hsa-miR-7151-3p, and hsa-miR-186-3p miRNAs targeting the *VHL* transcript in HPV-positive cells (Figure 8A), compared with HPV-negative cells. Notably, the miRNAs targeting the *PHD2* (*EGLN1*) transcript hsa-miR-6832-5p and hsa-miR-182-5p expression levels were high in HPV-positive cells compared to HPV-negative cells, and expression levels were statistically significant for hsa-miR-182-5p (Figure 8B). Specifically, miRNAs targeting the *CUL2* transcript were only expressed in hsa-miR-3179, where expression was lower in HPV-positive cells compared to HPV-negative cells (Figure 8C). On the other hand, high expression levels were observed for the miRNAs hsa-miR-618 and hsa-miR-7151-3p, which were transcriptionally targeted by *ELOC* in HPV-positive cells compared to HPV-negative cells (Figure 8D). These data demonstrate other possibilities in regulating the E3 ubiquitin ligase complex of VHL, CUL2, ELOC, and PHD2 hydroxylase by the effect of E6 and E7 of HPV 16.

**Figure 9:**
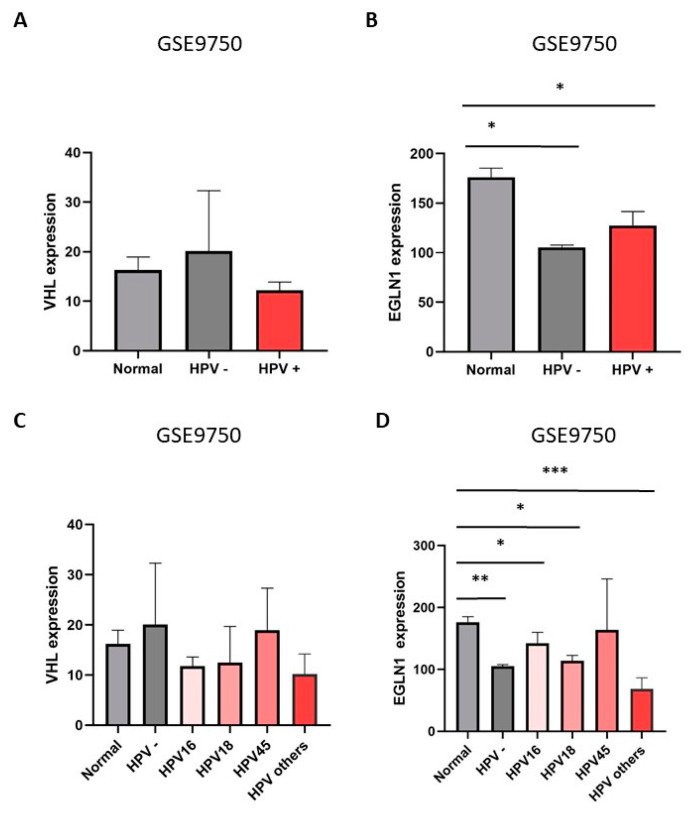
*VHL* and *PHD2* (*EGLN1*) expression in HPV-positive and HPV-negative CC patients. Expression levels of *VHL* (**A**) and *PHD2* (*EGLN1*) (**B**) in HPV-positive and HPV-negative CC patients. *VHL* and *PHD2* (*EGLN1*) expression was obtained from the GSE9750 dataset. (**C**,**D**) *VHL* and *PHD2* (*EGLN1*) expression using the GSE9750 dataset, including normal samples, CC samples positive for HPV genotypes 16, 18, 45 and other HPV genotypes. Fold change of *VHL* and *PHD2* (*EGLN1*) was analyzed in GraphPad Prism 8 software. Statistical significance: * *p* < 0.05, ** *p* < 0.01, *** *p* < 0.001.

Here, we found that the expression of hsa-miR-508-3p, hsa-miR-7151-3p, hsa-miR-186-3p, hsa-miR-6832-5p, hsa-miR-182-5p, hsa-miR-618, and hsa-miR-7151-3p was higher in HPV-positive CSCC and HNSCC cell lines. Our findings suggest that the presence of HPV is involved in the expression of miRNAs that negatively regulate the expression of the VCB-CR complex. In this regard, to validate whether *VHL* and *PHD2* (*EGLN1*) expression decreases in the presence of HPV, we analyzed the expression of *VHL* and *PHD2* (*EGLN1*) transcripts using the GEO dataset GSE9750, which includes samples from HPV-positive CC patients with the presence of HPV 16, 18, and 45, as well as HPV-negative genotypes and samples from normal cervical tissue (Figure 9). We also observed that *VHL* and *PHD2* (*EGLN1*) expression was lower in HPV-positive CC patients compared with normal cervical tissue (Figure 9A,B). Furthermore, in CC patients with HPV genotypes 16, 18, and 45 and other HPVs, the expression levels of *VHL* and *PHD2* (*EGLN1*) were low compared to normal cervical tissue samples (Figure 9C,D). These data suggest that the presence of HPV and HPV 16, 18, and 45 genotypes are involved in the expression of miRNAs hsa-miR-508-3p, hsa-miR-7151-3p, and hsa-miR-186-3p as well as in hsa-miR-6832-5p and hsa-miR-182-5p. Interestingly, these miRNAs displayed *VHL* and *PHD2* (*EGLN1*) as transcriptional targets, and consequently decrease the expression of *VHL* and *PHD2* (*EGLN1*) transcripts. Unfortunately, we did not find data related to the expression of CUL2 and ELOC from the database used.

### 3.7. Function Enrichment Analysis

**Figure 10:**
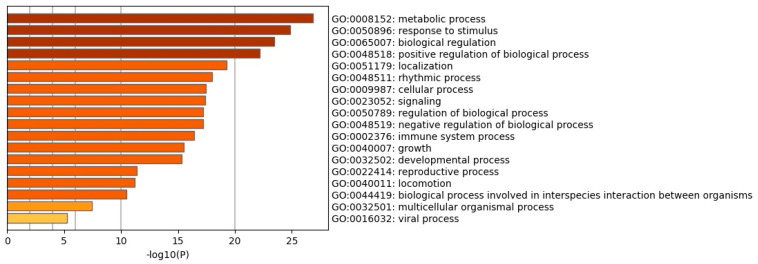
Function enrichment analysis of cellular processes altered by E6 and E7 related to HIF-1α dysregulation. Bar graph of enriched terms in the input list of genes showing physical, protein-DNA, and chemical interaction with HIF-1α. Bars are shown colored by −Log10(p).

**Figure 11:**
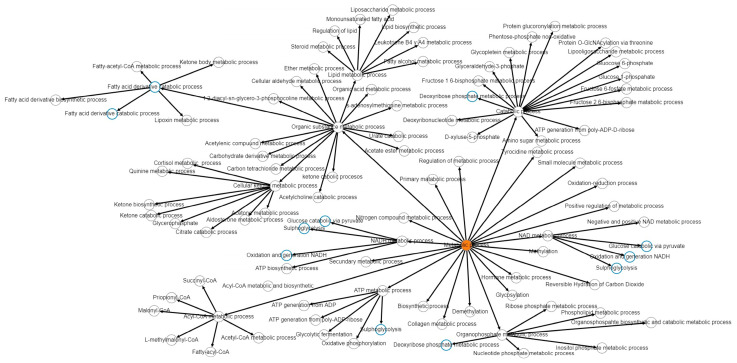
Network of metabolic pathways altered by HIF-1α. The analysis of the network of metabolic pathways regulated by HIF-1α shows the processes related to cellular metabolism. Among them are carbohydrate metabolism, organophosphate metabolism, NAD metabolism, NADH, ATP generation, Acyl-CoA metabolism, ketone and ketone body metabolism, organic substrate metabolism, and fatty acid metabolism.

Some reports indicate the role of VHL and HIF-1α in metabolic reprogramming [13,19,68,69,70,71]. For this reason, the possible effect that the E6 and E7 oncoproteins could have by interfering with the formation of the VHL, CUL2, and ELOC ubiquitin ligase complex that ubiquitinates HIF-1α was evaluated. From the list of proteins that have physical, protein/gene, and chemical interaction with HIF-1α, we proceeded to perform function and processes analysis by consulting GENEONTOLOGY, PANTHER, GOTERMFINDER, and Metascape, databases. The reliability parameters were <=0.001 for GOTERMFINDER and < 0.005 for GO and PANTHER. For Metascape, the top 20 function clusters were obtained, and the reliability values are represented as the −Log10(p). The data show that the processes that may be altered are mostly processes related to metabolic reprogramming −Log10(p) of 25 (Figure 10). Metabolism-related processes and functions were analyzed in PANTHER; the processes were integrated and shown in the metabolic pathway network generated in esyN (Figure 11).

### 3.8. Metabolic Changes Mediated by E6 and E7 of HPV 16 through Its Interactors and VHL-CUL2-ELOC-ELOB

**Figure 12:**
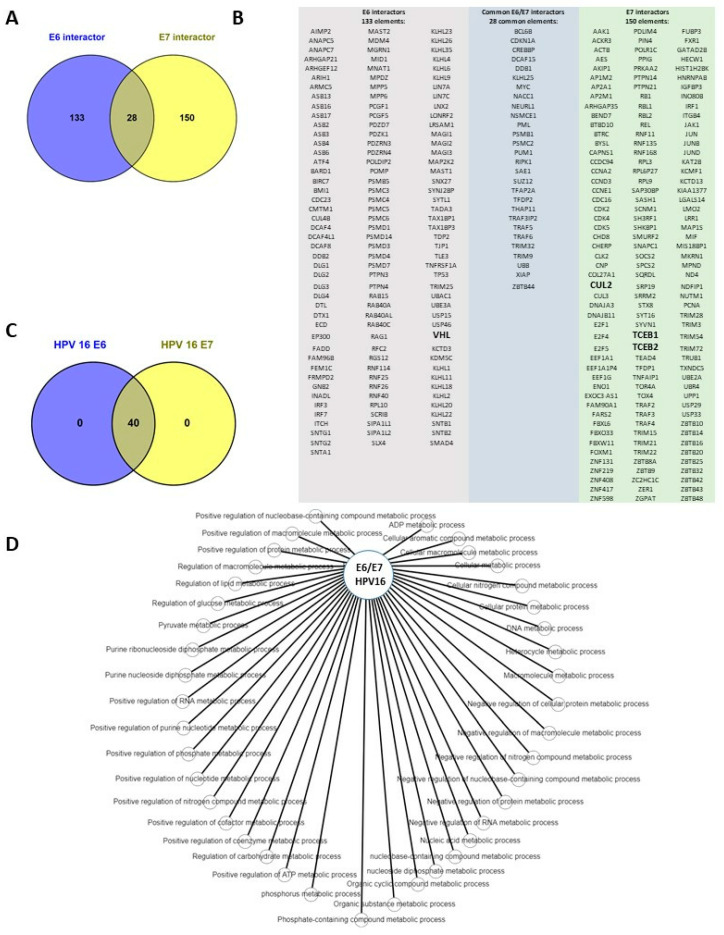
Metabolic changes mediated by the E6 and E7 oncoproteins of HPV 16. (**A**) VENN diagram of the common interactors and interactors of E6 and E7 of HPV 16. (**B**) A total of 133 interactors regulated by E6 (columns in gray), 150 interactors regulated by E7 (columns in green), and 28 interactors in common for E6 and E7 (columns in blue) are depicted. Bold names represent the interactors VHL, CUL2, TCEB1 (ELOC), and TCEB1 (ELOB). (**C**) VENN diagram of common metabolic processes and pathways regulated by E6 and E6; all 40 total metabolic processes are commonly regulated by E6 and E7. (**D**) Network of metabolic pathways altered by E6 and E7 of HPV 16. Data were obtained from the BioGRID database and the protein and gene list were used to introduce the PANTHER database.

To validate the metabolic changes in cancer cells by E7 and E7 oncoproteins, we analyzed the metabolic changes from the interactors, regulated by E6 and E7, which included VHL-CUL2-ELOC-ELOB. We also observed that E6 interacted with 161 proteins, whereas E7 interacted with 178; both oncoproteins interacted physically with their targets (Figure 12A). We also observed that of the total number of interactors, only 28 proteins had common interaction with E6 and E7 (Figure 12B). Interestingly, we observed in the analysis of metabolic changes that even though the proteins interacting with E6 and E7 were different, including the number, they regulated the same metabolic processes (Figure 12C), which highlights metabolic changes that were also observed and regulated by HIF-1α (Figure 11), such as lipid metabolism, ATP metabolism, ADP metabolism, carbohydrate metabolism (including glucose metabolism), and pyruvate metabolism. The total metabolic changes that were mediated by E6 and E7 are depicted in Figure 12D. We note that E6 and E7 through their targets including VHL-CUL2-ELOC-ELOB and HIF-1α are involved in tumor maintenance and progression, as well as energy production

## 4. Discussion

The oncogenic potential of HPV 16 is related to the production of E6 and E7 oncoproteins, which alter several processes related to cell transformation [72,73], including metabolic reprogramming, through degradation of p53 and retinoblastoma (pRb) and increased expression of c-Myc and HIF-1α, proteins that regulate cell metabolism in cancer [27].

In this study, the role of HPV 16 oncoproteins E6 and E7 in metabolic reprogramming, a hallmark of cancer, was analyzed [74]. Information on the interaction of E6 and E7 with VHL-CUL2-ELOC, proteins essential in the formation of the E3 ubiquitin ligase complex, responsible for the ubiquitination of HIF-1α for degradation via the proteasome, was searched through different databases [19]. In cancer, HIF-1α is stabilized, translocates to the nucleus, and binds to HIF-1β, forming the heterodimer and active transcription factor HIF-1, an essential regulator of metabolic reprogramming [75]. *HIF-1A* mRNA was overexpressed in HCC, HNSCC, and CRC samples compared to normal tissue. The development of CC is essentially dependent on an HPV infection of the cervix that must persist for many years and decades [76]. In HNSC, infection with HR-HPV has recently been implicated in the pathogenesis of this cancer. It is now widely accepted that high-risk HPV is the cause of almost all HNSCC [77]. In addition, the presence of HR-HPV has been frequently reported in the development of CRC [78,79]. Overall, we observed that *HIF1A* expression was not affected in the presence of HPV; however, we considered that HPV genotypes, including low-risk genotypes, could counteract *HIF1A* expression [80,81]. Interestingly, we observed that *HIF1A* expression was higher and statistically significant in the presence of HPV 45. Although we did not observe differences in *HIF1A* expression in patients with HPV 16, previous reports from our lab indicate that HPV 16 increases *HIF1A* expression and is viral copy number dependent [82] and that E6 and E7 of HPV 16 increase HIF-1α expression and stability [8]. In addition, we have reported that overexpression of E6 of HPV 16 increases the expression levels of the *HIF1A* transcript. Furthermore, *HIF1A* expression by E6 is differentially affected by HPV 16 E6 variants and have been oncogenic enhancers in cancer development through metabolic reprogramming [27]. These data agree with other reports where increased HIF-1α expression has been seen [83,84,85,86,87,88,89] and suggest that increased HIF-1α expression is associated with carcinogenesis by increasing the production of energy and biomass necessary for tumor formation. Likewise, it could be helpful as a prognostic marker in HCC, HNSC, and CRC.

E6 and E7 interact with different proteins related to cell transformation [90,91,92,93,94,95,96,97]. Interestingly, E6 and E7 were found to interact with four main proteins of the HIF-1α ubiquitination complex: VHL (UniProtKB—P40337), CUL2 (UniProtKB—Q13617), TCEB1 or ELOC (UniProtKB—Q15369), and TCEB2 or ELOB (UniProtKB—Q15370) [18,19]. E7 interacts with CUL2, ELOC, and ELOB, while E6 interacts with VHL. No direct interaction between E6, E7, and HIF-1α was observed. The interaction of E6 and E7 with the proteins that make up the E3 ubiquitin ligase complex, which participates in the ubiquitination and subsequent degradation of HIF-1α, could be causing dissociation of the ubiquitination complex and inactivation, as occurs in the dissociation of the E2F-pRb complex and inactivation of interferon regulatory factor-3 (IRF-3) [95,98]. In addition, E6 might be blocking the interaction of VHL with HIF-1α [57]. Previous reports demonstrate that E6 and E7 of HR-HPV alter glycolytic metabolism, pH regulation, oxidative phosphorylation, glutaminolysis, and TCA that is converted to an α-ketoacid intermediate: oxaloacetate, pyruvate, acetyl-CoA, or succinyl-CoA [99]. In addition, E6 of HPV 16 contributes to the HIF-1α-induced increase in glycolysis by attenuating the VHL-HIF-1α interaction, leading to increased lactate production and glucose consumption [57]. Poirson et al. report that E6 and E7 of HPV physically interact with proteins of the ubiquitin–proteasome system, where E6 interacts with VHL and E7 with CUL2 and ELOB [54]. Interaction with components of the ubiquitin–proteasome system, including those of the VCB-CR complex, leads to the regulation of different cellular processes, including metabolic reprogramming [100]. In this regard, E6 and E6 variants of HPV 16 have been described to increase glycolysis metabolism by enhancing the stability, nuclear localization, and transcriptional activity of HIF-1α [101].

E6 was observed to bind to VHL at the HIF-1α recognition site [54]. On the other hand, E7 binds to CUL2 and ELOC at the C3 domain located in the carboxy-terminal region [55,56]. ELOC binds to E7 through two BC boxes, and CUL2 binds through the CUL2 box [56]. Only interaction of E7 with ELOB was observed in the interactome; however, sequence analysis did not find the sequences and domains involved in this interaction.

The interaction of E6 with cellular proteins is through the PDZ domain, the name designated by the first three proteins that possess this domain Postsynaptic density protein (PSD95), Drosophila disc large tumor suppressor (Dlg1), and Zonula occludens-1 protein (ZO-1), which results in alterations in critical cellular functions such as cell polarity, signal transduction, and cell lifespan extension [102,103]. E6 of HPV 16 has the PDZ-binding motif (PBM), a specific sequence located at the carboxy-terminal end that interacts with PDZ-domain-containing proteins [58]. VHL was observed to have sequence homology to PDZ compared to the 58,648 proteins in the PDZ scape database library [46]. The predicted PDZ in VHL has a higher identity score (37.7) than the PDZ score of E6-AP (30.6). The predicted PDZ in VHL was observed to localize to the E6-interacting region. The data suggest that E7 may inhibit the formation of the E3 ubiquitin ligase complex by interacting with CUL2, ELOC, and ELOB proteins. In contrast, E6 may be blocking the recognition of HIF-1α by VHL, preventing its ubiquitination and subsequent degradation.

Epigenetically, HR-HPVs lead to aberrant expression of miRNAs [21,22]. Some of these miRNAs negatively regulate metabolism genes, involved in the tricarboxylic acid (TCA) cycle, aerobic glycolysis, de novo synthesis of fatty acids, and altered autophagy, which allow tumor cells to survive under adverse conditions. In addition, miRNAs may target signaling molecules such as HIF-1 that promote metabolic adaptation in tumor cells [23]. In addition, miRNAs have been reported to target *HIF1A*, *VHL*, *CUL*, *RBX1*, and *ELOC* [24,25,26]. Additionally, the E6 and E7 oncoproteins of HPV 16 lead to aberrant miRNA expression; a large number of these miRNA genes are downstream mRNA targets essential in normal cell maintenance [22,60]. In addition to looking for the effect of HPV 16 E6 and E7 oncoproteins on HIF-1α levels at the protein level, we looked for the possible effect of microRNAs (miRNAs) overexpressed in the presence of E6 and E7 on the mRNA level of genes coding for proteins of the PHD2-VHL-CUL2-ELOC axis [19]. We found 36 miRNAs targeting *VHL* mRNA, 19 targeting *PHD2*, 15 targeting *CUL2*, and 21 targeting *ELOC*, of which, hsa-miR-4326, hsa-miR-155-3p, hsa-miR-143-3p, hsa-miR-4714-5p, hsa-miR-508-3p, hsa-miR-29b-2-5p, hsa-miR-186-3p, and hsa-miR-7151-3p target *VHL*; hsa-miR-182-5p, hsa-miR-7-2-3p, hsa-miR-3128, hsa-miR-216a-5p, and hsa-miR-6832-5p target *PHD2*; hsa-miR-3179, hsa-miR-6129, hsa-miR-6127, hsa-miR-4510, and hsa-miR-297 target *CUL2*; and hsa-miR-1251-3p, hsa-miR-618, hsa-miR-208a-5p, hsa-miR-6762-3p, hsa-miR-7151-3p, hsa-miR-4451, and hsa-miR-504-3p target *ELOC*. These miRNAs had 52 to 99 Context score percentile, match in Position in the UTR, and 8mer as significant reliability parameters in the interaction of the miRNA seed region with the transcript of *PHD2*, *VHL*, *CUL2*, and *ELOC*. The E6 and E7 oncoproteins of HPV 16 are associated with increased expression of miRNAs, contributing to a rearrangement in gene expression and consequently to cell transformation in cancer [104,105]. We found that E6 and E7 increase hsa-miR-155-3p and hsa-miR-143-3p expression, two of the miRNAs that match with *VHL* [61,62,63], as well as the fact that E7 increases the levels of hsa-miR-182-5p, which in this analysis targets *PDH2* [64,65]. Of the miRNAs that regulate *CUL2*, experimental data demonstrated the expression of hsa-miR-6129 associated with HPV 16 [67]. Of the common miRNAs with high seed region complementarity scores for the *VHL*, *PHD2* (*EGLN1*), *CUL2*, and *ELOC* transcripts, we found high expression levels of three miRNAs targeting the *VHL* transcript, two for the *PHD2* (*EGLN1*) transcript and two for the *ELOC* transcript in HPV-positive cells. Interestingly, *VHL* and *PHD2* (*EGLN1*) expression levels are lower in HPV-positive and HPV 16-, 18-, and 45-positive CC patients. These data suggest that HPV and HPV 16, 18, and 45 genotypes are involved in the expression of miRNAs and, consequently, the lower expression levels of *VHL*, *PHD2* (*EGLN1*), and *ELOC*. In particular, miRNAs targeting *CUL2* transcript only obtained low expression data for one miRNA, and no *CUL2* expression data were found in the database. With these findings, we propose that E6 and E7 of HPV inhibit VCB-CR complex formation by physical interaction with VHL, CUL2, ELOC, and ELOB, as well as through the expression of miRNAs that block the expression of *VHL*, *PHD2* (*EGLN1*), and *ELOC* and consequently prevent VCB-CR complex formation.

The data found here suggest that E6 and E7 of HPV 16 could be interfering in the formation of the E3 ubiquitin ligase complex formed by VHL-CUL2-ELOC and at the post-transcriptional level as they also do with PHD2 by the expression of miRNAs. Consequently, HIF-1α is not ubiquitinated and is not sent to degradation, which may cause HIF-1α to bind to HIF-1β to form the active transcription factor HIF1, thus enabling transcription of its target genes. In this regard, the consequences of HIF-1α stability in cancer due to the effect of HPV 16 oncoproteins E6 and E7 involves the formation of active HIF-1 consisting of HIF-1α and HIF-1β, which is known to be an essential metabolic regulator that allows cancer cells to adapt to oxygen deprivation by enabling the transcription of 81 genes related to metabolic reprogramming [106]. In the interaction analysis, we reported 427 protein/gene interactions of HIF-1 with its targets. From the list of proteins interacting with HIF-1α, we observed that they mainly regulate metabolism-related processes such as PDP1 (pyruvate dehydrogenase phosphatase catalytic subunit 1), CA9 (carbonic anhydrase IX), PLD2 (phospholipase D2), ALDH1A3 (aldehyde dehydrogenase 1 family, member A3), LDHA (lactate dehydrogenase A), PFKFB4 (6-phosphofructo-2-kinase/fructose-2,6-biphosphatase 4), PKM (pyruvate kinase, muscle), GLUD1 (glutamate dehydrogenase 1), ATAD3A (ATPase family, AAA domain containing 3A), and BDH1 (3-hydroxybutyrate dehydrogenase, type 1), among others (Table S1). We also observed that of the metabolic processes that regulate the list of proteins whose expression is regulated by HIF-1α, the metabolic pathways in which it is crucial are glycolysis, pentose phosphate pathway, lipid metabolism, metabolism of ketone bodies, fatty acid synthesis, and obtaining nitrogenous compounds, all important in obtaining energy and biomass necessary for tumor formation and progression. These metabolic processes were also regulated by E6 and E7 of HPV 16. We suggest that these E6- and E7-regulated metabolic processes consistent with HIF-1α-regulated metabolic processes are due to the fact that together E6 and E7 synergistically regulate the VCB-CR complex. These findings are related to results previously found in our laboratory, where high expression of enzymes and transporters involved in glycolytic metabolism such as *SLC2A1* (*GLUT1*), *LDHA*, *SLC16A3* (*MCT4*), *CA9* (*CAIX*), and *BSG* (*Basigin* or *CD147*) were found to be associated with the grade of cervical intraepithelial neoplasia (CIN), invasive cervical carcinoma (ICC), and HPV 16 infection [82,107].

Interestingly, in clinical practice, these results propose evidence that HIF-1α and the products generated by HIF-1α-regulated metabolic processes could be employed as a metabolic signature in the diagnosis, prognosis, and treatment of HCC, HNSC, and non-invasive CRC, as it has been described in other types of cancer [108,109,110,111]. On the other hand, in cancer therapy directed against HIF-1α and VHL, it shows important effects, decreasing tumor growth and cancer progression [112]. Together, our data propose the usefulness of HIF-1α as a prognostic and/or predictive biomarker, as well as in personalized cancer therapy [113,114]. Although the results shown in this work were obtained from databases, it is important to emphasize that all the databases used in this bioinformatics analysis are fully supported and validated experimentally. The results of expression, survival, physical interaction, and enrichment pathways have been obtained by experimental studies ranging from RNA-Seq experiments, microarray for the case of expression, affinity capture-MS assays, FRET, Affinity Capture-Western, two-hybrid, co-localization, PCA a protein fragment complementation assay, and protein–peptide interaction, to mention some interaction assays. Specifically, in enrichment pathway analyses, the database algorithm comprises multiple sources of functional annotations for the given gene list, enriched in GO terms and encompassing enriched gene clusters, related to functions from multiple previously curated publications.

The role of HPV 16 oncoproteins E6 and E7 in metabolic reprogramming has been little studied. In this work, we propose a possible mechanism through which E6 and E7 participate in metabolic reprogramming in cancer by preventing the degradation of HIF-1α, the key subunit in forming the active transcription factor HIF-1. HIF-1 is a crucial transcription factor in regulating genes involved in energy metabolism and other processes important in cancer initiation and progression. These results suggest HIF-1α and its target genes as potential therapeutic targets or prognostic markers regulated by E6 and E7 of HPV 16 in CC, HNSC, and CCR.

## 5. Conclusions

In conclusion, this work presents the possible mechanism by which the E6 and E7 oncoproteins of HPV 16 could be affecting the PHD2-VHL-CUL2-ELOC-HIF-1α backbone and, consequently, metabolic reprogramming in CC, HNSC, and CRC cancers. Bioinformatics data suggesting that HIF-1α increases its expression in CC, HNSC, and CRC are presented. We suggest that the increase in HIF-1α levels is due to the interaction of E6 with VHL, possibly through a PDZ domain, as well as the interaction of E7 with CUL2 and ELOC, essential proteins of the E3 ubiquitin ligase complex through the carboxy-terminal domain of E7. E6 and E7 could interfere with the synergistic formation of the ubiquitin ligase complex that marks HIF-1α for degradation. E6 could be blocking the recognition of HIF-1α by VHL, whereas E7 could interfere with forming the ubiquitin ligase complex by interacting with CUL2 and ELOC. In addition, the miRNAs that at the post-transcriptional level could be regulating gene expression of the PHD2-VHL-CUL2 and ELOC backbone are shown. Finally, the effect of E6 and E7 on metabolic processes such as carbohydrate metabolism; organophosphate metabolism; NAD, NADH, and ATP metabolism; Acyl-CoA metabolism; ketone body metabolism; organic substrate metabolism; and fatty acid metabolism, which are altered in metabolic reprogramming by affecting hydroxylation, ubiquitination, and degradation of HIF-1α, is shown.

## Figures and Tables

**Table 1:** Major miRNAs that bind to *VHL*, *PHD2*, *CUL2*, and *ELOC* mRNAs.

mARNs	miRNAs	Position in the UTR	Seed Match	Context Score Percentile
*VHL*	hsa-miR-4326	520–527	8mer	97
	hsa-miR-155-3p	646–653		97
	hsa-miR-143-3p	27–34		98
	hsa-miR-4714-5p	109–116		99
	hsa-miR-508-3p	1540–1547		95
	hsa-miR-29b-2-5p	3470–3477		99
	hsa-miR-186-3p	421–428		66
	hsa-miR-7151-3p	1838–1845		52
*PHD2*	hsa-miR-182-5p	1644–1651	8mer	98
	hsa-miR-7-2-3p	48–55		87
	hsa-miR-3128	301–308		99
	hsa-miR-216a-5p	1023–1030		62
	hsa-miR-6832-5p	1968–1975		79
*CUL2*	hsa-miR-3179	278–285	8mer	98
	hsa-miR-6129	1211–1218		94
	hsa-miR-6127	1211–1218		91
	hsa-miR-4510	1211–1218		91
	hsa-miR-297	1219–1226		92
*ELOC*	hsa-miR-1251-3p	120–127	8mer	99
	hsa-miR-618	408–415		99
	hsa-miR-208a-5p	836–843		97
	hsa-miR-6762-3p	1044–1051		99
	hsa-miR-7151-3p	1232–1239		95
	hsa-miR-4451	1325–1332		98
	hsa-miR-504-3p	1458–1465		92

## Data Availability

The data generated in the present study may be requested from the corresponding author.

## Supplementary Materials

The following supporting information can be downloaded at https://www.mdpi.com/article/10.3390/cimb46060370/s1. Table S1: Functions of HIF-1α molecular targets.
