# Supplementary material for: Bioinformatics Analysis Reveals E6 and E7 of HPV 16 Regulate Metabolic Reprogramming in Cervical Cancer, Head and Neck Cancer, and Colorectal Cancer through the PHD2-VHL-CUL2-ELOC-HIF-1α Axis
Source: Curr Issues Mol Biol. 2024 Jun 19;46(6):6199–222. doi: 10.3390/cimb46060370 (PMC11202971; doi:10.3390/cimb46060370)
Supplement: Supplementary file 1 [file cimb-46-00370-s001.zip › cimb-3016979-supplementary.pdf]

Supplementary Table S1. Functions of HIF-1α Molecular Targets.

Interactor	Organism	Aliases	Description
MATR3	H. sapiens	MPD2, ALS21, VCPDM	matrin 3
CDK2	H. sapiens	CDKN2, p33(CDK2)	cyclin-dependent kinase 2
HDAC2	H. sapiens	HD2, YAF1, RPD3	histone deacetylase 2
XRCC5	H. sapiens	KUB2, NFIV, KU80, Ku86, KARP1, KARP-1	X-ray repair complementing defective repair in Chinese hamster cells 5 (double-strand-break rejoining)
RUNX1	H. sapiens	AML1, CBFA2, EVI-1, AMLCR1, PEBP2aB, AML1-EVI-1	runt-related transcription factor 1
HNRNPF	H. sapiens	HNRPF, mcs94-1, OK/SW-cl.23	heterogeneous nuclear ribonucleoprotein F
MSH2	H. sapiens	FCC1, HNPCC, COCA1, LCFS2, HNPCC1	mutS homolog 2
SMARCA4	H. sapiens	BRG1, SWI2, SNF2, RTPS2, MRD16, SNF2L4, SNF2LB, BAF190, hSNF2b, BAF190A	SWI/SNF related, matrix associated, actin dependent regulator of chromatin, subfamily a, member 4
TWIST1	H. sapiens	CRS, CSO, SCS, ACS3, CRS1, BPES3, BPES2, TWIST, bHLHa38	twist family bHLH transcription factor 1
PCBP1	H. sapiens	HNRPX, HNRPE1, hnRNP-X, HEL-S-85, hnRNP-E1	poly(rC) binding protein 1
RUVBL2	H. sapiens	RVB2, TIH2, ECP51, TIP48, REPTIN, TIP49B, INO80J, CGI-46	RuvB-like AAA ATPase 2
MYL1	H. sapiens	MLC3F, MLC1F	myosin, light chain 1, alkali; skeletal, fast
ING1	H. sapiens	p33, p47, p33ING1, p47ING1a, p24ING1c, p33ING1b, RP11-8D7.1	inhibitor of growth family, member 1
CREBBP	H. sapiens	CBP, RSTS, KAT3A	CREB binding protein
TRIM21	H. sapiens	SSA, SSA1, RO52, RNF81, Ro/SSA	tripartite motif containing 21
MARCH7	H. sapiens	AXO, AXOT, RNF177, MARCH-VII	membrane-associated ring finger (C3HC4) 7, E3 ubiquitin protein ligase
FASN	H. sapiens	FAS, OA-519, SDR27X1	fatty acid synthase
FLII	H. sapiens	FLI, FLIL, Fli1	flightless I homolog (Drosophila)
CDC34	H. sapiens	UBC3, UBCH3, UBE2R1, E2-CDC34	cell division cycle 34
HDAC6	H. sapiens	HD6, JM21, CPBHM, PPP1R90	histone deacetylase 6
APEX1	H. sapiens	APE, APX, APE1, HAP1, APEX, REF1, APEN	APEX nuclease (multifunctional DNA repair enzyme) 1
MYH2	H. sapiens	IBM3, MYH2A, MYHas8, MYHSA2, MyHC-2A, MyHC-IIa	myosin, heavy chain 2, skeletal muscle, adult
HIST1H4A	H. sapiens	H4FA	histone cluster 1, H4a
EPAS1	H. sapiens	HLF, MOP2, HIF2A, ECYT4, PASD2, bHLHe73	endothelial PAS domain protein 1
PGK1	H. sapiens	PGKA, MIG10, HEL-S-68p, RP4-570L12.1	phosphoglycerate kinase 1
RUNX2	H. sapiens	CCD, OSF2, CLCD, AML3, CCD1, CBFA1, OSF-2, PEA2aA, PEBP2aA, RP1-166H4.1, ... more	runt-related transcription factor 2
ATM	H. sapiens	ATD, ATE, ATC, ATA, AT1, TEL1, ATDC, TELO1	ATM serine/threonine kinase
CDK4	H. sapiens	CMM3, PSK-J3	cyclin-dependent kinase 4
TBCD	H. sapiens	tfcD, SSD-1, PP1096	tubulin folding cofactor D
FAF2	H. sapiens	ETEA, UBXD8, UBXN3B	Fas associated factor family member 2
ARRB2	H. sapiens	ARR2, ARB2, BARR2	arrestin, beta 2
MAP2K5	H. sapiens	MEK5, PRKMK5, MAPKK5, HsT17454	mitogen-activated protein kinase kinase 5
NTPCR	H. sapiens	C1orf57, HCR-NTPase, RP4-659I19.2	nucleoside-triphosphatase, cancer-related
TCEB2	H. sapiens	ELOB, SIII	transcription elongation factor B (SIII), polypeptide 2 (18kDa, elongin B)

UTP23	H. sapiens	C8orf53	UTP23, small subunit (SSU) processome component, homolog (yeast)
PDP1	H. sapiens	PDP, PDH, PDPC, PPM2C	pyruvate dehyrogenase phosphatase catalytic subunit 1
JUN	H. sapiens	AP1, AP-1, c-Jun	jun proto-oncogene
FBXO8	H. sapiens	FBS, DC10, FBX8	F-box protein 8
MKRN2	H. sapiens	RNF62, HSPC070	makorin ring finger protein 2
SIRT1	H. sapiens	SIR2L1, RP11-57G10.3	sirtuin 1
BAG2	H. sapiens	BAG-2, dJ417I1.2, RP3-496N17.2	BCL2-associated athanogene 2
LINC01139	H. sapiens	-	long intergenic non-protein coding RNA 1139
TTC1	H. sapiens	TPR1	tetratricopeptide repeat domain 1
LRPPRC	H. sapiens	LSFC, GP130, LRP130, CLONE-23970	leucine-rich pentatricopeptide repeat containing
ESRRB	H. sapiens	ERR2, ERRb, ESRL2, NR3B2, DFNB35	estrogen-related receptor beta
SSX4	H. sapiens	CT5.4, RP11-344N17.14-001	synovial sarcoma, X breakpoint 4
HIST1H4I	H. sapiens	H4M, H4/m, H4FM	histone cluster 1, H4i
RFC2	H. sapiens	RFC40	replication factor C (activator 1) 2, 40kDa
CDKN2A	H. sapiens	P19, ARF, MLM, P14, P16, CMM2, MTS1, INK4, TP16, INK4A, ... more	cyclin-dependent kinase inhibitor 2A
CDK8	H. sapiens	K35	cyclin-dependent kinase 8
RUNX3	H. sapiens	AML2, CBFA3, PEBP2aC, RP3-398I9.1	runt-related transcription factor 3
MAX	H. sapiens	bHLHd4	MYC associated factor X
CREB1	H. sapiens	CREB	cAMP responsive element binding protein 1
NSFL1C	H. sapiens	P47, UBX1, UBXD10, UBXN2C, dJ776F14.1, RP4-776F14.2	NSFL1 (p97) cofactor (p47)
MAFK	H. sapiens	P18, NFE2U	v-maf avian musculoaponeurotic fibrosarcoma oncogene homolog K
DCAF8	H. sapiens	GAN2, H326, WDR42A, RP11-574F21.1	DDB1 and CUL4 associated factor 8
GNB2L1	H. sapiens	HLC7, HLC-7, H12.3, PIG21, RACK1, Gnb2-rs1	guanine nucleotide binding protein (G protein), beta polypeptide 2-like 1
PIN1	H. sapiens	DOD, UBL5	peptidylprolyl cis/trans isomerase, NIMA-interacting 1
DNAJB6	H. sapiens	DJ4, MRJ, HSJ2, DnaJ, MSJ-1, HSJ-2, HHDJ1, LGMD1E, LGMD1D, tcag7.555	DnaJ (Hsp40) homolog, subfamily B, member 6
HNRNPM	H. sapiens	CEAR, HTGR1, HNRPM, NAGR1, HNRPM4, HNRNPM4, hnRNP M	heterogeneous nuclear ribonucleoprotein M
KDM4C	H. sapiens	GASC1, JMJD2C, JHDM3C, TDRD14C, bA146B14.1, RP11-169L18.1	lysine (K)-specific demethylase 4C
VHLL	H. sapiens	VLP, VHLP	von Hippel-Lindau tumor suppressor-like
MED13L	H. sapiens	THRAP2, TRAP240L, PROSIT240	mediator complex subunit 13-like
ELK1	H. sapiens	-	ELK1, member of ETS oncogene family
CDK6	H. sapiens	MCPH12, PLSTIRE	cyclin-dependent kinase 6
NDN	H. sapiens	PWCR, HsT16328	necdin, melanoma antigen (MAGE) family member
FTCD	H. sapiens	LCHC1	formimidoyltransferase cyclodeaminase
KEAP1	H. sapiens	INrf2, KLHL19	kelch-like ECH-associated protein 1
BHLHE41	H. sapiens	DEC2, hDEC2, BHLHB3, SHARP1	basic helix-loop-helix family, member e41
PSMA7	H. sapiens	C6, HSPC, XAPC7, RC6-1, RP5-1005F21.4	proteasome (prosome, macropain) subunit, alpha type, 7
HIF1A	H. sapiens	HIF1, MOP1, PASD8, HIF-1A, bHLHe78, HIF1-ALPHA, HIF-1alpha	hypoxia inducible factor 1, alpha subunit (basic helix-loop-helix transcription factor)

RBM38	H. sapiens	RNPC1, SEB4D, SEB4B, HSRNASEB, dJ800J21.2	RNA binding motif protein 38
PLK3	H. sapiens	CNK, FNK, PRK, RP11-269F19.6	polo-like kinase 3
HSPA2	H. sapiens	HSP70-2, HSP70-3	heat shock 70kDa protein 2
ICAM1	H. sapiens	BB2, CD54, P3.58	intercellular adhesion molecule 1
LRRK2	H. sapiens	ROCO2, PARK8, RIPK7, AURA17, DARDARIN	leucine-rich repeat kinase 2
MED14	H. sapiens	CSRP, RGR1, EXLM1, CRSP2, CXorf4, TRAP170, CRSP150, DRIP150	mediator complex subunit 14
DNAI1	H. sapiens	PCD, DIC1, ICS1, CILD1, RP11-296L22.2	dynein, axonemal, intermediate chain 1
UBXN1	H. sapiens	2B28, SAKS1, UBXD10	UBX domain protein 1
RAB13	H. sapiens	GIG4	RAB13, member RAS oncogene family
UBXN7	H. sapiens	UBXD7	UBX domain protein 7
H2AFX	H. sapiens	H2AX, H2A/X, H2A.X	H2A histone family, member X
CREB3L1	H. sapiens	OASIS, PSEC0238	cAMP responsive element binding protein 3-like 1
UBE3C	H. sapiens	HECTH2, tcag7.998	ubiquitin protein ligase E3C
SPTLC1	H. sapiens	SPTI, LBC1, LCB1, SPT1, HSN1, HSAN1, RP11-118F2.1	serine palmitoyltransferase, long chain base subunit 1
KPNA5	H. sapiens	SRP6, IPOA6, RP3-412I7.4	karyopherin alpha 5 (importin alpha 6)
FBXW7	H. sapiens	AGO, FBW7, FBW6, hAgo, CDC4, hCdc4, FBX30, SEL10, FBXW6, SEL-10, ... more	F-box and WD repeat domain containing 7, E3 ubiquitin protein ligase
ACTR2	H. sapiens	ARP2	ARP2 actin-related protein 2 homolog (yeast)
VWA8	H. sapiens	KIAA0564, RP11-125A7.3	von Willebrand factor A domain containing 8
CDC6	H. sapiens	HsCDC6, CDC18L, HsCDC18	cell division cycle 6
DNAJA1	H. sapiens	HSDJ, DjA1, DJ-2, HDJ2, HSJ2, HSPF4, NEDD7, hDJ-2, RP11-54K16.1	DnaJ (Hsp40) homolog, subfamily A, member 1
YAP1	H. sapiens	YKI, YAP, YAP2, COB1, YAP65	Yes-associated protein 1
RNF5	H. sapiens	RMA1, RING5, DAAP-218M18.2	ring finger protein 5, E3 ubiquitin protein ligase
MDM2	H. sapiens	HDMX, hdm2, ACTFS	MDM2 proto-oncogene, E3 ubiquitin protein ligase
SIN3A	H. sapiens	-	SIN3 transcription regulator family member A
RHOBTB3	H. sapiens	-	Rho-related BTB domain containing 3
SUCO	H. sapiens	OPT, CH1, SLP1, C1orf9	SUN domain containing ossification factor
MAP2K3	H. sapiens	MEK3, MKK3, PRKMK3, MAPKK3, SAPKK2, SAPKK-2	mitogen-activated protein kinase kinase 3
MYH3	H. sapiens	SMHCE, HEMHC, MYHSE1, MYHC-EMB	myosin, heavy chain 3, skeletal muscle, embryonic
MYH6	H. sapiens	ASD3, SSS3, MYHC, CMH14, MYHCA, CMD1EE, alpha-MHC	myosin, heavy chain 6, cardiac muscle, alpha
FBXL16	H. sapiens	Fbl16, C16orf22, c380A1.1	F-box and leucine-rich repeat protein 16
CCAR2	H. sapiens	DBC1, DBC-1, NET35, p30DBC, p30 DBC, KIAA1967	cell cycle and apoptosis regulator 2
KLHL8	H. sapiens	-	kelch-like family member 8
USP19	H. sapiens	ZMYND9	ubiquitin specific peptidase 19
WWTR1	H. sapiens	TAZ	WW domain containing transcription regulator 1
NBEAL1	H. sapiens	ALS2CR17, ALS2CR16, A530083I02Rik	neurobeachin-like 1
TUFM	H. sapiens	P43, EFTU, COXPD4, EF-TuMT	Tu translation elongation factor, mitochondrial
SMYD3	H. sapiens	KMT3E, ZMYND1, ZNFN3A1, bA74P14.1, RP11-49H10.1	SET and MYND domain containing 3
MED15	H. sapiens	TIG1, CTG7A, CAG7A, TIG-1, PCQAP, TNRC7, ARC105	mediator complex subunit 15

PCGF2	H. sapiens	RNF110, MEL-18, ZNF144	polycomb group ring finger 2
PFKL	H. sapiens	PFK-B, PFK-L, ATP-PFK	phosphofructokinase, liver
KPNA6	H. sapiens	KPNA7, IPOA7, RP4-622L5.1	karyopherin alpha 6 (importin alpha 7)
RNF4	H. sapiens	SLX5, SNURF, RES4-26	ring finger protein 4
PRKACA	H. sapiens	PKACA	protein kinase, cAMP-dependent, catalytic, alpha
TET1	H. sapiens	LCX, CXXC6, bA119F7.1, RP11-119F7.1	tet methylcytosine dioxygenase 1
DNAJA3	H. sapiens	TID1, HCA57, hTID-1	DnaJ (Hsp40) homolog, subfamily A, member 3
PSMA4	H. sapiens	HC9, PSC9, HsT17706	proteasome (prosome, macropain) subunit, alpha type, 4
FHL1	H. sapiens	KYOT, SLIM, FHL1A, FHL1B, FLH1A, XMPMA, FHL-1, SLIM1, SLIM-1, SLIMMER, ... more	four and a half LIM domains 1
PTBP1	H. sapiens	PTB, pPTB, PTB4, PTB2, PTB3, PTB-1, PTB-T, HNRPI, HNRNPI, HNRNP-I	polypyrimidine tract binding protein 1
KPNA1	H. sapiens	RCH2, SRP1, IPOA5, NPI-1	karyopherin alpha 1 (importin alpha 5)
SAT1	H. sapiens	SAT, DC21, SSAT, KFSD, KFSDX, SSAT-1	spermidine/spermine N1-acetyltransferase 1
STK39	H. sapiens	SPAK, PASK, DCHT	serine threonine kinase 39
CX3CR1	H. sapiens	V28, GPR13, CCRL1, GPRV28, CMKDR1, CMKBRL1	chemokine (C-X3-C motif) receptor 1
RORA	H. sapiens	RZRA, ROR3, ROR2, ROR1, NR1F1, RZR-ALPHA	RAR-related orphan receptor A
GATA3	H. sapiens	HDR, HDRS	GATA binding protein 3
IKBKAP	H. sapiens	FD, DYS, IKAP, TOT1, IKI3, ELP1, RP11-3J11.4	inhibitor of kappa light polypeptide gene enhancer in B-cells, kinase complex-associated protein
FBXO28	H. sapiens	Fbx28, CENP-30	F-box protein 28
HNRNPL	H. sapiens	HNRPL, hnRNP-L, P/OKcl.14	heterogeneous nuclear ribonucleoprotein L
UBC	H. sapiens	HMG20	ubiquitin C
HIF1AN	H. sapiens	FIH1	hypoxia inducible factor 1, alpha subunit inhibitor
UACA	H. sapiens	NUCLING	uveal autoantigen with coiled-coil domains and ankyrin repeats
PLD1	H. sapiens	-	phospholipase D1, phosphatidylcholine-specific
CDK1	H. sapiens	CDC2, CDC28A, P34CDC2	cyclin-dependent kinase 1
STK11	H. sapiens	PJS, LKB1, hLKB1	serine/threonine kinase 11
BCR	H. sapiens	ALL, CML, PHL, BCR1, D22S11, D22S662	breakpoint cluster region
PYCARD	H. sapiens	ASC, TMS, TMS1, CARD5, TMS-1	PYD and CARD domain containing
RHBDF1	H. sapiens	Dist1, hDist1, gene-89, gene-90, EGFR-RS, C16orf8	rhomboid 5 homolog 1 (Drosophila)
AKT1	H. sapiens	RAC, AKT, PKB, CWS6, PRKBA, RAC-ALPHA, PKB-ALPHA	v-akt murine thymoma viral oncogene homolog 1
KPNA4	H. sapiens	QIP1, SRP3, IPOA3	karyopherin alpha 4 (importin alpha 3)
GTF2I	H. sapiens	WBS, SPIN, DIWS, IB291, BTKAP1, TFII-I, BAP135, WBSCR6, GTFII-I	general transcription factor IIi
SQSTM1	H. sapiens	p60, p62, p62B, PDB3, OSIL, A170, ZIP3	sequestosome 1
RACGAP1	H. sapiens	CYK4, ID-GAP, HsCYK-4, MgcRacGAP	Rac GTPase activating protein 1
ISG15	H. sapiens	G1P2, IP17, UCRP, hUCRP, IFI15, IMD38	ISG15 ubiquitin-like modifier
NANOG	H. sapiens	-	Nanog homeobox
PPP2R1A	H. sapiens	PR65A, PP2AAALPHA, PP2A-Aalpha	protein phosphatase 2, regulatory subunit A, alpha
EGLN3	H. sapiens	PHD3, HIFPH3, HIFP4H3	egl-9 family hypoxia-inducible factor 3

RPTOR	H. sapiens	KOG1, Mip1	regulatory associated protein of MTOR, complex 1
DCUN1D1	H. sapiens	SCRO, RP42, Tes3, SCCRO, DCNL1, DCUN1L1	DCN1, defective in cullin neddylation 1, domain containing 1
MED13	H. sapiens	THRAP1, ARC250, HSPC221, TRAP240, DRIP250	mediator complex subunit 13
ARF3	H. sapiens	-	ADP-ribosylation factor 3
HSP90AA1	H. sapiens	EL52, LAP2, HSPN, Hsp90, HSPC1, LAP-2, HSP86, HSPCA, Hsp89, HSP90N, ... more	heat shock protein 90kDa alpha (cytosolic), class A member 1
CUL2	H. sapiens	RP11-297A16.3	cullin 2
NAA10	H. sapiens	TE2, ARD1, NATD, ARD1P, ARD1A, DXS707, MCOPS1	N(alpha)-acetyltransferase 10, NatA catalytic subunit
TRIM28	H. sapiens	TF1B, KAP1, TIF1B, RNF96, PPP1R157	tripartite motif containing 28
LYSMD2	H. sapiens	-	LysM, putative peptidoglycan-binding, domain containing 2
FDX1	H. sapiens	FDX, ADX, LOH11CR1D	ferredoxin 1
USP25	H. sapiens	USP21	ubiquitin specific peptidase 25
PFKFB3	H. sapiens	PFK2, IPFK2, RP11-298K24.3	6-phosphofructo-2-kinase/fructose-2,6-biphosphatase 3
MSH6	H. sapiens	p160, HSAP, GTBP, GTMBP, HNPCC5	mutS homolog 6
RUVBL1	H. sapiens	RVB1, TIH1, ECP54, TIP49, PONTIN, TIP49A, NMP238, INO80H, Pontin52	RuvB-like AAA ATPase 1
IQGAP1	H. sapiens	SAR1, p195, HUMORFA01	IQ motif containing GTPase activating protein 1
SMARCA2	H. sapiens	BRM, hBRM, SWI2, SNF2, Sth1p, NCBRS, SNF2L2, SNF2LA, BAF190, hSNF2a	SWI/SNF related, matrix associated, actin dependent regulator of chromatin, subfamily a, member 2
SUMO1	H. sapiens	UBL1, PIC1, SMT3, DAP1, GMP1, OFC10, SMT3C, SENP2, SMT3H3, OK/SW-cl.43	small ubiquitin-like modifier 1
NES	H. sapiens	Nbla00170	nestin
CA9	H. sapiens	MN, CAIX	carbonic anhydrase IX
SRPRB	H. sapiens	APMCF1, PSEC0230	signal recognition particle receptor, B subunit
EP300	H. sapiens	p300, RSTS2, KAT3B, RP1-85F18.1	E1A binding protein p300
ARRB1	H. sapiens	ARR1, ARB1	arrestin, beta 1
PLEKHA5	H. sapiens	PEPP2, PEPP-2	pleckstrin homology domain containing, family A member 5
PLD2	H. sapiens	-	phospholipase D2
ESRRG	H. sapiens	ERR3, NR3B3, ERRgamma	estrogen-related receptor gamma
DDX39A	H. sapiens	DDXL, BAT1, URH49, DDX39, BAT1L	DEAD (Asp-Glu-Ala-Asp) box polypeptide 39A
FN1	H. sapiens	FN, CIG, FNZ, MSF, ED-B, GFND, LETS, FINC, GFND2	fibronectin 1
GPLD1	H. sapiens	PLD, PIGPLD, GPIPLD, GPIPLDM, PIGPLD1	glycosylphosphatidylinositol specific phospholipase D1
ALDH1A3	H. sapiens	ALDH6, MCOP8, RALDH3, ALDH1A6, RP11-66B24.1	aldehyde dehydrogenase 1 family, member A3
RFC5	H. sapiens	RFC36	replication factor C (activator 1) 5, 36.5kDa
XPO5	H. sapiens	exp5, RP3-337H4.5	exportin 5
RWDD3	H. sapiens	RSUME	RWD domain containing 3
MIF	H. sapiens	GIF, GLIF, MMIF	macrophage migration inhibitory factor (glycosylation-inhibiting factor)
MYH4	H. sapiens	MYH2B, MyHC-2B, MyHC-IIb	myosin, heavy chain 4, skeletal muscle
HSPA4	H. sapiens	RY, HSPH2, APG-2, hsp70, hsp70RY, HS24/P52, HEL-S-5a	heat shock 70kDa protein 4
TEAD2	H. sapiens	ETF, TEF4, TEF-4, TEAD-2	TEA domain family member 2
WWOX	H. sapiens	FOR, WOX1, FRA16D, SCAR12, HHCMA56, PRO0128, SDR41C1, D16S432E	WW domain containing oxidoreductase

EGLN2	H. sapiens	EIT6, PHD1, HPH-3, HPH-1, HIFPH1, HIF-PH1	egl-9 family hypoxia-inducible factor 2
EPO	H. sapiens	EP, MVCD2	erythropoietin
JUP	H. sapiens	DP3, PDGB, PKGB, CTNNG, DPIII, ARVD12	junction plakoglobin
BCL2	H. sapiens	Bcl-2, PPP1R50	B-cell CLL/lymphoma 2
sep-04	H. sapiens	H5, ARTS, MART, SEP4, CE5B3, PNUTL2, hucep-7, BRADEION, hCDCREL-2	septin 4
MYO1B	H. sapiens	myr1	myosin IB
KIAA0368	H. sapiens	ECM29, RP11-386D8.2	KIAA0368
METTL15	H. sapiens	METT5D1	methyltransferase like 15
SETD7	H. sapiens	KMT7, SET7, SET9, SET7/9	SET domain containing (lysine methyltransferase) 7
LDHA	H. sapiens	LDHM, LDH1, PIG19, GSD11, HEL-S-133P	lactate dehydrogenase A
IKBKB	H. sapiens	IKKB, IKK2, IMD15, NFKBIKB, IKK-beta	inhibitor of kappa light polypeptide gene enhancer in B-cells, kinase beta
ZC3H12A	H. sapiens	MCPIP, MCPIP1, dJ423B22.1, RP3-423B22.1	zinc finger CCCH-type containing 12A
CXCR4	H. sapiens	LCR1, WHIM, HM89, LAP3, FB22, NPYR, LAP-3, NPYRL, NPY3R, LESTR, ... more	chemokine (C-X-C motif) receptor 4
TMEM33	H. sapiens	SHINC3, 1600019D15Rik	transmembrane protein 33
IPO5	H. sapiens	imp5, Pse1, IMB3, KPNB3, RANBP5, RP11-72J7.1	importin 5
FAT1	H. sapiens	ME5, FAT, CDHR8, CDHF7, hFat1	FAT atypical cadherin 1
SF3B2	H. sapiens	Cus1, SF3b1, SAP145, SF3b150, SF3B145	splicing factor 3b, subunit 2, 145kDa
DAP3	H. sapiens	DAP-3, MRPS29, bMRP-10, MRP-S29	death associated protein 3
TGFB1	H. sapiens	CED, LAP, TGFB, DPD1, TGFbeta	transforming growth factor, beta 1
GBF1	H. sapiens	ARF1GEF	golgi brefeldin A resistant guanine nucleotide exchange factor 1
SNHG11	H. sapiens	C20orf198, LINC00101, NCRNA00101	small nucleolar RNA host gene 11 (non-protein coding)
HSPA1L	H. sapiens	hum70t, HSP70T, HSP70-1L, HSP70-HOM, DADB-333F21.5	heat shock 70kDa protein 1-like
SP1	H. sapiens	-	Sp1 transcription factor
KIAA1429	H. sapiens	MSTP054, fSAP121	KIAA1429
PKM	H. sapiens	TCB, PK3, PKM2, OIP3, CTHBP, THBP1, HEL-S-30	pyruvate kinase, muscle
RICTOR	H. sapiens	PIA, AVO3, hAVO3	RPTOR independent companion of MTOR, complex 2
HSPA8	H. sapiens	LAP1, NIP71, LAP-1, HSC70, HSC71, HSC54, HSP71, HSP73, HEL-33, HSPA10, ... more	heat shock 70kDa protein 8
RFC4	H. sapiens	A1, RFC37	replication factor C (activator 1) 4, 37kDa
HSD17B12	H. sapiens	KAR, SDR12C1	hydroxysteroid (17-beta) dehydrogenase 12
NR4A1	H. sapiens	N10, HMR, TR3, NP10, GFRP1, NAK-1, NGFIB, NUR77	nuclear receptor subfamily 4, group A, member 1
VEGFA	H. sapiens	VPF, VEGF, MVCD1, RP1-261G23.1	vascular endothelial growth factor A
HIF1A-AS2	H. sapiens	aHIF, 3'aHIF-1A	HIF1A antisense RNA 2
GLUD1	H. sapiens	GDH, GLUD, GDH1	glutamate dehydrogenase 1
EARS2	H. sapiens	MSE1, COXPD12	glutamyl-tRNA synthetase 2, mitochondrial
ESRRA	H. sapiens	ERR1, ERRa, ESRL1, NR3B1, ERRalpha	estrogen-related receptor alpha
MYO9B	H. sapiens	MYR5, CELIAC4	myosin IXB
KPNA3	H. sapiens	SRP4, SRP1, hSRP1, IPOA4, SRP1gamma, RP11-432M24.3	karyopherin alpha 3 (importin alpha 4)
ARL1	H. sapiens	ARFL1	ADP-ribosylation factor-like 1

HEATR1	H. sapiens	BAP28, UTP10, RP11-385F5.3	HEAT repeat containing 1
USP9X	H. sapiens	FAF, FAM, MRX99, DFFRX, RP5-1172N10.4	ubiquitin specific peptidase 9, X-linked
ZNF197	H. sapiens	P18, VHLaK, ZNF20, ZNF166, ZSCAN41, ZKSCAN9, D3S1363E	zinc finger protein 197
POLR2B	H. sapiens	RPB2, POL2RB, hRPB140	polymerase (RNA) II (DNA directed) polypeptide B, 140kDa
HNF4A	H. sapiens	TCF, MODY, HNF4, FRTS4, TCF14, MODY1, NR2A1, HNF4a9, HNF4a8, HNF4a7, ... more	hepatocyte nuclear factor 4, alpha
USP29	H. sapiens	HOM-TES-84/86	ubiquitin specific peptidase 29
PSMD10	H. sapiens	p28, p28(GANK), dJ889N15.2, RP5-889N15.3	proteasome (prosome, macropain) 26S subunit, non-ATPase, 10
ALDH1B1	H. sapiens	ALDH5, ALDHX	aldehyde dehydrogenase 1 family, member B1
TXNIP	H. sapiens	THIF, VDUP1, HHCPA78, EST01027, RP11-315I20.4	thioredoxin interacting protein
KLF6	H. sapiens	GBF, ZF9, PAC1, CPBP, BCD1, CBA1, ST12, COPEB, RP11-184A2.1	Kruppel-like factor 6
UBE2D1	H. sapiens	SFT, UBCH5, UBC4/5, UBCH5A, E2(17)KB1	ubiquitin-conjugating enzyme E2D 1
TRAF6	H. sapiens	RNF85, MGC:3310	TNF receptor-associated factor 6, E3 ubiquitin protein ligase
HDAC4	H. sapiens	HD4, BDMR, AHO3, HDACA, HDAC-A, HA6116, HDAC-4	histone deacetylase 4
STAT3	H. sapiens	HIES, APRF, ADMIO	signal transducer and activator of transcription 3 (acute-phase response factor)
HK1	H. sapiens	HKD, HKI, HXK1, HMSNR, HK1-tc, HK1-tb, HK1-ta	hexokinase 1
MED12	H. sapiens	OKS, OPA1, FGS1, HOPA, OHDOX, ARC240, TNRC11, MED12S, CAGH45, TRAP230	mediator complex subunit 12
USP20	H. sapiens	VDU2, hVDU2, LSFR3A	ubiquitin specific peptidase 20
LATS2	H. sapiens	KPM	large tumor suppressor kinase 2
VASP	H. sapiens	-	vasodilator-stimulated phosphoprotein
CCND2	H. sapiens	MPPH3, KIAK0002	cyclin D2
SAT2	H. sapiens	SSAT2	spermidine/spermine N1-acetyltransferase family member 2
ERAL1	H. sapiens	ERA, H-ERA, HERA-B, HERA-A, ERAL1A	Era-like 12S mitochondrial rRNA chaperone 1
SART3	H. sapiens	p110, P100, DSAP1, TIP110, p110(nrb), RP11-13G14	squamous cell carcinoma antigen recognized by T cells 3
ABCF2	H. sapiens	ABC28, HUSSY18, HUSSY-18, EST133090	ATP-binding cassette, sub-family F (GCN20), member 2
TP73	H. sapiens	P73	tumor protein p73
TUBA1A	H. sapiens	LIS3, TUBA3, B-ALPHA-1	tubulin, alpha 1a
NSF	H. sapiens	SKD2	N-ethylmaleimide-sensitive factor
CEBPA	H. sapiens	CEBP, C/EBP-alpha	CCAAT/enhancer binding protein (C/EBP), alpha
COMMD1	H. sapiens	MURR1, C2orf5	copper metabolism (Murr1) domain containing 1
COPS5	H. sapiens	JAB1, CSN5, SGN5, MOV-34	COP9 signalosome subunit 5
FOS	H. sapiens	p55, AP-1, C-FOS	FBJ murine osteosarcoma viral oncogene homolog
EFTUD2	H. sapiens	MFDM, MFDGA, Snu114, Snrp116, U5-116KD, SNRNP116	elongation factor Tu GTP binding domain containing 2
ELP3	H. sapiens	KAT9	elongator acetyltransferase complex subunit 3
NCOA1	H. sapiens	SRC1, RIP160, KAT13A, bHLHe74, F-SRC-1, bHLHe42	nuclear receptor coactivator 1
SMAD3	H. sapiens	LDS3, MADH3, LDS1C, JV15-2, HSPC193, HsT17436	SMAD family member 3

CAND1	H. sapiens	TIP120, TIP120A	cullin-associated and neddylation-dissociated 1
SIRT2	H. sapiens	SIR2, SIR2L, SIR2L2	sirtuin 2
HEXIM1	H. sapiens	CLP1, MAQ1, HIS1, EDG1	hexamethylene bis-acetamide inducible 1
EPHA2	H. sapiens	ECK, CTPA, CTPP1, ARCC2, CTRCT6	EPH receptor A2
VCP	H. sapiens	p97, TERA, ALS14, IBMPFD, HEL-220, IBMPFD1, HEL-S-70	valosin containing protein
FZR1	H. sapiens	FZR, FZR2, HCDH, CDH1, HCDH1, CDC20C	fizzy/cell division cycle 20 related 1 (Drosophila)
IDH3B	H. sapiens	RP46, H-IDHB, RP4-686C3.2	isocitrate dehydrogenase 3 (NAD+) beta
EGLN1	H. sapiens	HPH2, PHD2, SM20, HPH-2, ECYT3, ZMYND6, HIFPH2, HIF-PH2, C1orf12, PNAS-118	egl-9 family hypoxia-inducible factor 1
RLIM	H. sapiens	RNF12, NY-REN-43, CTD-2530H13.3	ring finger protein, LIM domain interacting
UMPS	H. sapiens	OPRT, OK/SW-cl.21	uridine monophosphate synthetase
CASR	H. sapiens	FHH, CAR, HHC, FIH, EIG8, HHC1, PCAR1, NSHPT, GPRC2A, HYPOC1	calcium-sensing receptor
ESR1	H. sapiens	ER, ESR, Era, ESRA, ESTRR, NR3A1, RP1-130E4.1	estrogen receptor 1
ACTA1	H. sapiens	NEM3, MPFD, CFTD, ASMA, NEM1, NEM2, ACTA, CFTDM, CFTD1, RP5-1068B5.2	actin, alpha 1, skeletal muscle
ATAD3A	H. sapiens	RP5-832C2.1	ATPase family, AAA domain containing 3A
RUNX1T1	H. sapiens	CDR, ETO, MTG8, AML1T1, ZMYND2, CBFA2T1	runt-related transcription factor 1; translocated to, 1 (cyclin D-related)
IQGAP3	H. sapiens	-	IQ motif containing GTPase activating protein 3
CHD4	H. sapiens	CHD-4, Mi-2b, Mi2-BETA	chromodomain helicase DNA binding protein 4
USP28	H. sapiens	-	ubiquitin specific peptidase 28
PARK2	H. sapiens	PDJ, PRKN, LPRS2, AR-JP, KB-152G3.1	parkin RBR E3 ubiquitin protein ligase
FGFR4	H. sapiens	TKF, JTK2, CD334	fibroblast growth factor receptor 4
PFKFB4	H. sapiens	-	6-phosphofructo-2-kinase/fructose-2,6-biphosphatase 4
DDB1	H. sapiens	XPE, XPCE, DDBA, XAP1, XPE-BF, UV-DDB1	damage-specific DNA binding protein 1, 127kDa
DPM1	H. sapiens	MPDS, CDGIE, RP5-914P20.2	dolichyl-phosphate mannosyltransferase polypeptide 1, catalytic subunit
BTAF1	H. sapiens	MOT1, TAF172, TAFII170, TAF(II)170	BTAF1 RNA polymerase II, B-TFIID transcription factor-associated, 170kDa
BNIP3	H. sapiens	NIP3	BCL2/adenovirus E1B 19kDa interacting protein 3
RELA	H. sapiens	p65, NFKB3	v-rel avian reticuloendotheliosis viral oncogene homolog A
PTGES3	H. sapiens	P23, TEBP, cPGES	prostaglandin E synthase 3 (cytosolic)
TCEB1	H. sapiens	eloC, SIII	transcription elongation factor B (SIII), polypeptide 1 (15kDa, elongin C)
MED23	H. sapiens	SUR2, CRSP3, MRT18, SUR-2, ARC130, CRSP130, CRSP133, DRIP130, RP5-914N13.2	mediator complex subunit 23
MAPK1	H. sapiens	p38, p40, p41, ERK, ERK2, ERT1, ERK-2, PRKM1, MAPK2, PRKM2, ... more	mitogen-activated protein kinase 1
PTK6	H. sapiens	BRK	protein tyrosine kinase 6
CAPN1	H. sapiens	CANP, muCL, CANP1, PIG30, muCANP, CANPL1	calpain 1, (mu/I) large subunit
CEP350	H. sapiens	GM133, CAP350, RP11-502H18.1	centrosomal protein 350kDa
AIFM1	H. sapiens	AIF, CMTX4, NAMSD, COWCK, CMT2D, NADMR, PDCD8, COXPD6, RP3-438D16.2	apoptosis-inducing factor, mitochondrion-associated, 1
BDH1	H. sapiens	BDH, SDR9C1	3-hydroxybutyrate dehydrogenase, type 1

NEDD8	H. sapiens	NEDD-8	neural precursor cell expressed, developmentally down-regulated 8
HDAC3	H. sapiens	HD3, RPD3, RPD3-2	histone deacetylase 3
CTNNB1	H. sapiens	CTNNB, MRD19, armadillo, OK/SW-cl.35	catenin (cadherin-associated protein), beta 1, 88kDa
CDK9	H. sapiens	TAK, CTK1, C-2k, CDC2L4, PITALRE, RP11-228B15.5	cyclin-dependent kinase 9
sep-09	H. sapiens	MSF, NAPB, MSF1, SINT1, PNUTL4, SeptD1, AF17q25	septin 9
GLIS2	H. sapiens	NKL, NPHP7	GLIS family zinc finger 2
CDKN2B	H. sapiens	P15, MTS2, TP15, CDK4I, INK4B, p15INK4b, RP11-149I2.1	cyclin-dependent kinase inhibitor 2B (p15, inhibits CDK4)
IKBKG	H. sapiens	IP, IP2, IP1, NEMO, FIP3, IPD2, Fip3p, FIP-3, IMD33, ZC2HC9, ... more	inhibitor of kappa light polypeptide gene enhancer in B-cells, kinase gamma
MCM7	H. sapiens	MCM2, CDC47, P85MCM, PNAS146, P1CDC47, PPP1R104, P1.1-MCM3	minichromosome maintenance complex component 7
NUP210	H. sapiens	GP210, POM210, PSEC0245	nucleoporin 210kDa
HECTD1	H. sapiens	EULIR	HECT domain containing E3 ubiquitin protein ligase 1
YBX1	H. sapiens	YB1, DBPB, BP-8, YB-1, CSDB, CSDA2, NSEP1, NSEP-1, MDR-NF1	Y box binding protein 1
MTOR	H. sapiens	FRAP, RAPT1, RAFT1, FRAP1, FRAP2	mechanistic target of rapamycin (serine/threonine kinase)
TP63	H. sapiens	p51, p63, NBP, RHS, LMS, AIS, p40, KET, EEC3, p73H, ... more	tumor protein p63
POLRMT	H. sapiens	MTRNAP, MTRPOL, APOLMT, h-mtRPOL	polymerase (RNA) mitochondrial (DNA directed)
XPOT	H. sapiens	XPO3	exportin, tRNA
HCFC1	H. sapiens	CFF, HCF, MRX3, VCAF, HCF1, HFC1, HCF-1, PPP1R89	host cell factor C1
RALY	H. sapiens	P542, HNRPCL2, RP1-64K7.1	RALY heterogeneous nuclear ribonucleoprotein
EIF5A2	H. sapiens	eIF5AII, EIF-5A2	eukaryotic translation initiation factor 5A2
EAF2	H. sapiens	U19, BM040, TRAITS, BM-040	ELL associated factor 2
CBL	H. sapiens	CBL2, NSLL, RNF55, C-CBL, FRA11B	Cbl proto-oncogene, E3 ubiquitin protein ligase
SHC1	H. sapiens	SHC, SHCA, RP11-307C12.1	SHC (Src homology 2 domain containing) transforming protein 1
PSMC3	H. sapiens	TBP1	proteasome (prosome, macropain) 26S subunit, ATPase, 3
HDAC7	H. sapiens	HD7A, HDAC7A	histone deacetylase 7
PRKDC	H. sapiens	HYRC, p350, DNAPK, HYRC1, DNPK1, IMD26, XRCC7, DNA-PKcs	protein kinase, DNA-activated, catalytic polypeptide
SAMHD1	H. sapiens	DCIP, HDDC1, MOP-5, CHBL2, SBBI88, RP1-132F21.1	SAM domain and HD domain 1
NBN	H. sapiens	NBS, P95, ATV, NBS1, AT-V2, AT-V1	nibrin
ARNT	H. sapiens	HIF1B, TANGO, bHLHe2, HIF1BETA, HIF-1beta, HIF1-beta, HIF-1-beta	aryl hydrocarbon receptor nuclear translocator
RB1	H. sapiens	RB, pRb, OSRC, pp110, p105-Rb, PPP1R130, RP11-174I10.1	retinoblastoma 1
OS9	H. sapiens	OS-9, ERLEC2	osteosarcoma amplified 9, endoplasmic reticulum lectin
USP22	H. sapiens	USP3L	ubiquitin specific peptidase 22
KAT5	H. sapiens	TIP, PLIP, ESA1, TIP60, cPLA2, ZC2HC5, HTATIP, HTATIP1	K(lysine) acetyltransferase 5
HIST1H2BH	H. sapiens	H2BFJ, H2B/j	histone cluster 1, H2bh
CTSB	H. sapiens	APPS, CPSB	cathepsin B
E2F7	H. sapiens	-	E2F transcription factor 7
USP7	H. sapiens	TEF1, HAUSP	ubiquitin specific peptidase 7 (herpes virus-associated)

CPEB2	H. sapiens	CPEB-2, CPE-BP2, hCPEB-2	cytoplasmic polyadenylation element binding protein 2
HDAC5	H. sapiens	HD5, NY-CO-9	histone deacetylase 5
DCAF13	H. sapiens	GM83, WDSOF1, HSPC064	DDB1 and CUL4 associated factor 13
IL11	H. sapiens	AGIF, IL-11	interleukin 11
STUB1	H. sapiens	CHIP, UBOX1, SCAR16, NY-CO-7, SDCCAG7, HSPABP2, LA16c-313D11.6	STIP1 homology and U-box containing protein 1, E3 ubiquitin protein ligase
PARP1	H. sapiens	PARP, PPOL, ADPRT, ARTD1, PARP-1, ADPRT1, ADPRT 1, pADPRT-1, RP11-125A15.2	poly (ADP-ribose) polymerase 1
PNPLA6	H. sapiens	sws, NTE, BNHS, SPG39, NTEMND, iPLA2delta	patatin-like phospholipase domain containing 6
KAT2B	H. sapiens	CAF, PCAF, P/CAF	K(lysine) acetyltransferase 2B
BRCA1	H. sapiens	IRIS, PSCP, FANCS, RNF53, BRCC1, PNCA4, BRCAI, PPP1R53, BROVCA1	breast cancer 1, early onset
TRIP13	H. sapiens	16E1BP	thyroid hormone receptor interactor 13
CBWD1	H. sapiens	COBP, RP11-143M1.6	COBW domain containing 1
NLN	H. sapiens	MEP, MOP, AGTBP, EP24.16	neurolysin (metallopeptidase M3 family)
CSNK1D	H. sapiens	ASPS, HCKID, FASPS2, CKIdelta	casein kinase 1, delta
RAD23B	H. sapiens	P58, HR23B, HHR23B, RP11-131A5.1	RAD23 homolog B (S. cerevisiae)
ELL	H. sapiens	MEN, ELL1, PPP1R68, C19orf17	elongation factor RNA polymerase II
ARNT2	H. sapiens	WEDAS, bHLHe1	aryl-hydrocarbon receptor nuclear translocator 2
MID2	H. sapiens	FXY2, TRIM1, RNF60, MRX101, RP6-191P20.2	midline 2
EP400	H. sapiens	P400, CAGH32, TNRC12	E1A binding protein p400
HSP90AB1	H. sapiens	HSPC2, HSP84, HSPCB, D6S182, HSP90B, RP1-302G2.1	heat shock protein 90kDa alpha (cytosolic), class B member 1
KDM3A	H. sapiens	TSGA, JMJD1, JHDM2A, JHMD2A, JMJD1A	lysine (K)-specific demethylase 3A
PDGFRB	H. sapiens	IMF1, IBGC4, PDGFR, JTK12, CD140B, PDGFR1, PDGFR-1	platelet-derived growth factor receptor, beta polypeptide
DNAJA2	H. sapiens	DJ3, RDJ2, DJA2, DNJ3, DNAJ, CPR3, HIRIP4, PRO3015	DnaJ (Hsp40) homolog, subfamily A, member 2
NQO1	H. sapiens	DTD, QR1, DHQU, DIA4, NMOR1, NMORI	NAD(P)H dehydrogenase, quinone 1
SRC	H. sapiens	ASV, SRC1, c-SRC, p60-Src, RP5-823N20.1	SRC proto-oncogene, non-receptor tyrosine kinase
PFKM	H. sapiens	PFKX, PFK1, GSD7, PFKA, PFK-1, ATP-PFK, PPP1R122	phosphofructokinase, muscle
OTUB1	H. sapiens	OTU1, OTB1, HSPC263	OTU deubiquitinase, ubiquitin aldehyde binding 1
GKAP1	H. sapiens	GKAP42, FKSG21	G kinase anchoring protein 1
PELO	H. sapiens	CGI-17, PRO1770	pelota homolog (Drosophila)
MMS19	H. sapiens	MET18, MMS19L, hMMS19, RP11-452K12.5	MMS19 nucleotide excision repair homolog (S. cerevisiae)
CLP1	H. sapiens	HEAB, hClp1	cleavage and polyadenylation factor I subunit 1
NUP93	H. sapiens	NIC96	nucleoporin 93kDa
CBFA2T3	H. sapiens	ETO2, MTG16, MTGR2, ZMYND4	core-binding factor, runt domain, alpha subunit 2; translocated to, 3
SMURF2	H. sapiens	-	SMAD specific E3 ubiquitin protein ligase 2
AURKA	H. sapiens	AIK, BTAK, AURA, STK7, STK6, ARK1, STK15, PPP1R47, AURORA2, RP5-1167H4.6	aurora kinase A
SLC25A22	H. sapiens	GC1, NET44, EIEE3	solute carrier family 25 (mitochondrial carrier: glutamate), member 22
FOXM1	H. sapiens	TGT3, MPP2, HFH11, MPP-2, INS-1, HNF-3, PIG29, FKHL16, FOXM1B, HFH-11, ... more	forkhead box M1

UNC45A	H. sapiens	SMAP1, SMAP-1, GCUNC45, UNC-45A, GCUNC-45, GC-UNC45, IRO039700	unc-45 homolog A (C. elegans)
GNL3	H. sapiens	NS, E2IG3, NNP47, C77032	guanine nucleotide binding protein-like 3 (nucleolar)
MAFG	H. sapiens	hMAF	v-maf avian musculoaponeurotic fibrosarcoma oncogene homolog G
VHL	H. sapiens	VHL1, pVHL, RCA1, HRCA1	von Hippel-Lindau tumor suppressor, E3 ubiquitin protein ligase
MCM3	H. sapiens	P1.h, RLFB, HCC5, P1-MCM3, RP1-108C2.3	minichromosome maintenance complex component 3
FAF1	H. sapiens	hFAF1, CGI-03, HFAF1s, UBXD12, UBXN3A	Fas (TNFRSF6) associated factor 1
PPP1R13L	H. sapiens	RAI, RAI4, IASPP, NKIP1	protein phosphatase 1, regulatory subunit 13 like
HDAC1	H. sapiens	HD1, RPD3, RPD3L1, GON-10, RP4-811H24.2	histone deacetylase 1
C19ORF70	H. sapiens	QIL1, P117	chromosome 19 open reading frame 70
SIRT6	H. sapiens	SIR2L6	sirtuin 6
AR	H. sapiens	KD, TFM, AIS, SBMA, DHTR, NR3C4, HYSP1, SMAX1, HUMARA, RP11-383C12.1	androgen receptor
NF2	H. sapiens	SCH, ACN, BANF	neurofibromin 2 (merlin)
RBX1	H. sapiens	ROC1, RNF75, BA554C12.1, RP11-554C12.1	ring-box 1, E3 ubiquitin protein ligase
SENP3	H. sapiens	Ulp1, SSP3, SMT3IP1	SUMO1/sentrin/SMT3 specific peptidase 3
MTA1	H. sapiens	-	metastasis associated 1
ELAVL1	H. sapiens	HUR, Hua, MelG, ELAV1	ELAV like RNA binding protein 1
DNAJB1	H. sapiens	Sis1, Hdj1, Hsp40, HSPF1, RSPH16B	DnaJ (Hsp40) homolog, subfamily B, member 1
ZNF518A	H. sapiens	ZNF518	zinc finger protein 518A
PRPF6	H. sapiens	TOM, ANT1, Prp6, RP60, ANT-1, hPrp6, U5-102K, C20orf14, SNRNP102	pre-mRNA processing factor 6
PIAS1	H. sapiens	GBP, ZMIZ3, DDXBP1, GU/RH-II	protein inhibitor of activated STAT, 1
XRCC6	H. sapiens	ML8, KU70, TLAA, CTC75, CTCBF, G22P1, CTA-216E10.7	X-ray repair complementing defective repair in Chinese hamster cells 6
FGF11	H. sapiens	FHF3	fibroblast growth factor 11
HIF3A	H. sapiens	MOP7, IPAS, PASD7, HIF-3A, bHLHe17	hypoxia inducible factor 3, alpha subunit
MYH1	H. sapiens	MYHa, HEL71, MYHSA1, MyHC-2x, MyHC-2X/D	myosin, heavy chain 1, skeletal muscle, adult
SNRNP200	H. sapiens	RP33, BRR2, HELIC2, ASCC3L1, U5-200KD	small nuclear ribonucleoprotein 200kDa (U5)
OTUD7B	H. sapiens	ZA20D1, CEZANNE, RP11-212K13.2	OTU deubiquitinase 7B
MON2	H. sapiens	-	MON2 homolog (S. cerevisiae)
F12	H. sapiens	HAF, HAE3, HAEX	coagulation factor XII (Hageman factor)
TCEANC2	H. sapiens	C1orf83, RP4-758J24.3	transcription elongation factor A (SII) N-terminal and central domain containing 2
TIMM50	H. sapiens	TIM50, TIM50L, PRO1512	translocase of inner mitochondrial membrane 50 homolog (S. cerevisiae)
PCBP2	H. sapiens	HNRPE2, HNRNPE2, hnRNP-E2	poly(rC) binding protein 2
GCN1L1	H. sapiens	GCN1, GCN1L, PRIC295	GCN1 general control of amino-acid synthesis 1-like 1 (yeast)
SPRY2	H. sapiens	hSPRY2	sprouty homolog 2 (Drosophila)
MCL1	H. sapiens	TM, EAT, MCL1S, Mcl-1, MCL1L, BCL2L3, MCL1-ES, mcl1/EAT, bcl2-L-3	myeloid cell leukemia 1
TRIM37	H. sapiens	MUL, POB1, TEF3	tripartite motif containing 37
TP53	H. sapiens	P53, LFS1, BCC7, TRP53	tumor protein p53
MYC	H. sapiens	MYCC, MRTL, c-Myc, bHLHe39	v-myc avian myelocytomatosis viral oncogene homolog

FHL3	H. sapiens	SLIM2	four and a half LIM domains 3
ETV4	H. sapiens	E1AF, PEA3, PEAS3, E1A-F	ets variant 4
FANCI	H. sapiens	KIAA1794	Fanconi anemia, complementation group I
BANP	H. sapiens	SMAR1, BEND1, SMARBP1	BTG3 associated nuclear protein
TRRAP	H. sapiens	Tra1, TR-AP, PAF400, STAF40, PAF350/400	transformation/transcription domain-associated protein
CYP1A2	H. sapiens	CP12, P3-450, P450(PA)	cytochrome P450, family 1, subfamily A, polypeptide 2
FHL2	H. sapiens	DRAL, AAG11, SLIM3, FHL-2, SLIM-3	four and a half LIM domains 2
RPL4	H. sapiens	L4	ribosomal protein L4
DIMT1	H. sapiens	DIM1, HUSSY5, DIMT1L, HSA9761, HUSSY-05	DIM1 dimethyladenosine transferase 1 homolog (S. cerevisiae)
XPO1	H. sapiens	emb, CRM1, exp1	exportin 1
RFC3	H. sapiens	RFC38	replication factor C (activator 1) 3, 38kDa
UCHL1	H. sapiens	PGP95, PARK5, NDGOA, PGP9.5, Uch-L1, HEL-117, PGP 9.5	ubiquitin carboxyl-terminal esterase L1 (ubiquitin thiolesterase)
ACTL6A	H. sapiens	Arp4, ACTL6, BAF53A, INO80K, ARPN-BETA	actin-like 6A
LAMP2	H. sapiens	LAMPB, LAMP-2, LGP110, CD107b	lysosomal-associated membrane protein 2
PRPF8	H. sapiens	RP13, PRP8, PRPC8, HPRP8, SNRNP220	pre-mRNA processing factor 8
HMGA1	H. sapiens	HMG-R, HMGIY, HMGA1A, RP11-513I15.2	high mobility group AT-hook 1
PDK1	H. sapiens	-	pyruvate dehydrogenase kinase, isozyme 1
UBE2I	H. sapiens	P18, UBC9, C358B7.1, LA16c-358B7.1	ubiquitin-conjugating enzyme E2I
CPSF1	H. sapiens	P/cl.18, CPSF160, HSU37012	cleavage and polyadenylation specific factor 1, 160kDa
HNRNPF	H. sapiens	HNRPF, mcs94-1, OK/SW-cl.23	heterogeneous nuclear ribonucleoprotein F
